# Integration of Single-Cell Transcriptomics With a High Throughput Functional Screening Assay to Resolve Cell Type, Growth Kinetics, and Stemness Heterogeneity Within the Comma-1D Cell Line

**DOI:** 10.3389/fgene.2022.894597

**Published:** 2022-06-14

**Authors:** Arpit Dave, Erin Nekritz, Daniel Charytonowicz, Michael Beaumont, Melissa Smith, Kristin Beaumont, Jose Silva, Robert Sebra

**Affiliations:** ^1^ Department of Genetics and Genomic Sciences, Icahn School of Medicine at Mount Sinai, New York, NY, United States; ^2^ Department of Pathology, Icahn School of Medicine at Mount Sinai Hospital, New York, NY, United States; ^3^ Icahn Genomics Institute, Icahn School of Medicine at Mount Sinai, New York, NY, United States; ^4^ Department of Biochemistry and Molecular Genetics, University of Louisville, Louisville, KY, United States; ^5^ Black Family Stem Cell Institute, Icahn School of Medicine at Mount Sinai, New York, NY, United States; ^6^ Center for Advanced Genomics Technology, Icahn School of Medicine at Mount Sinai, New York, NY, United States; ^7^ Sema4, A Mount Sinai Venture, Stamford, CT, United States

**Keywords:** scRNASeq, Mammary Development, Comma-1D, Mouse Cell Line, Functional Assay, High throughput

## Abstract

Cell lines are one of the most frequently implemented model systems in life sciences research as they provide reproducible high throughput testing. Differentiation of cell cultures varies by line and, in some cases, can result in functional modifications within a population. Although research is increasingly dependent on these *in vitro* model systems, the heterogeneity within cell lines has not been thoroughly investigated. Here, we have leveraged high throughput single-cell assays to investigate the Comma-1D mouse cell line that is known to differentiate in culture. Using scRNASeq and custom single-cell phenotype assays, we resolve the clonal heterogeneity within the referenced cell line on the genomic and functional level. We performed a cohesive analysis of the transcriptome of 5,195 sequenced cells, of which 85.3% of the total reads successfully mapped to the mm10-3.0.0 reference genome. Across multiple gene expression analysis pipelines, both luminal and myoepithelial lineages were observed. Deep differential gene expression analysis revealed eight subclusters identified as luminal progenitor, luminal differentiated, myoepithelial differentiated, and fibroblast subpopulations—suggesting functional clustering within each lineage. Gene expression of published mammary stem cell (MaSC) markers Epcam, Cd49f, and Sca-1 was detected across the population, with 116 (2.23%) sequenced cells expressing all three markers. To gain insight into functional heterogeneity, cells with patterned MaSC marker expression were isolated and phenotypically investigated through a custom single-cell high throughput assay. The comparison of growth kinetics demonstrates functional heterogeneity within each cell cluster while also illustrating significant limitations in current cell isolation methods. We outlined the upstream use of our novel automated cell identification platform—to be used prior to single-cell culture—for reduced cell stress and improved rare cell identification and capture. Through compounding single-cell pipelines, we better reveal the heterogeneity within Comma-1D to identify subpopulations with specific functional characteristics.

## Introduction

**FIGURE 1:**
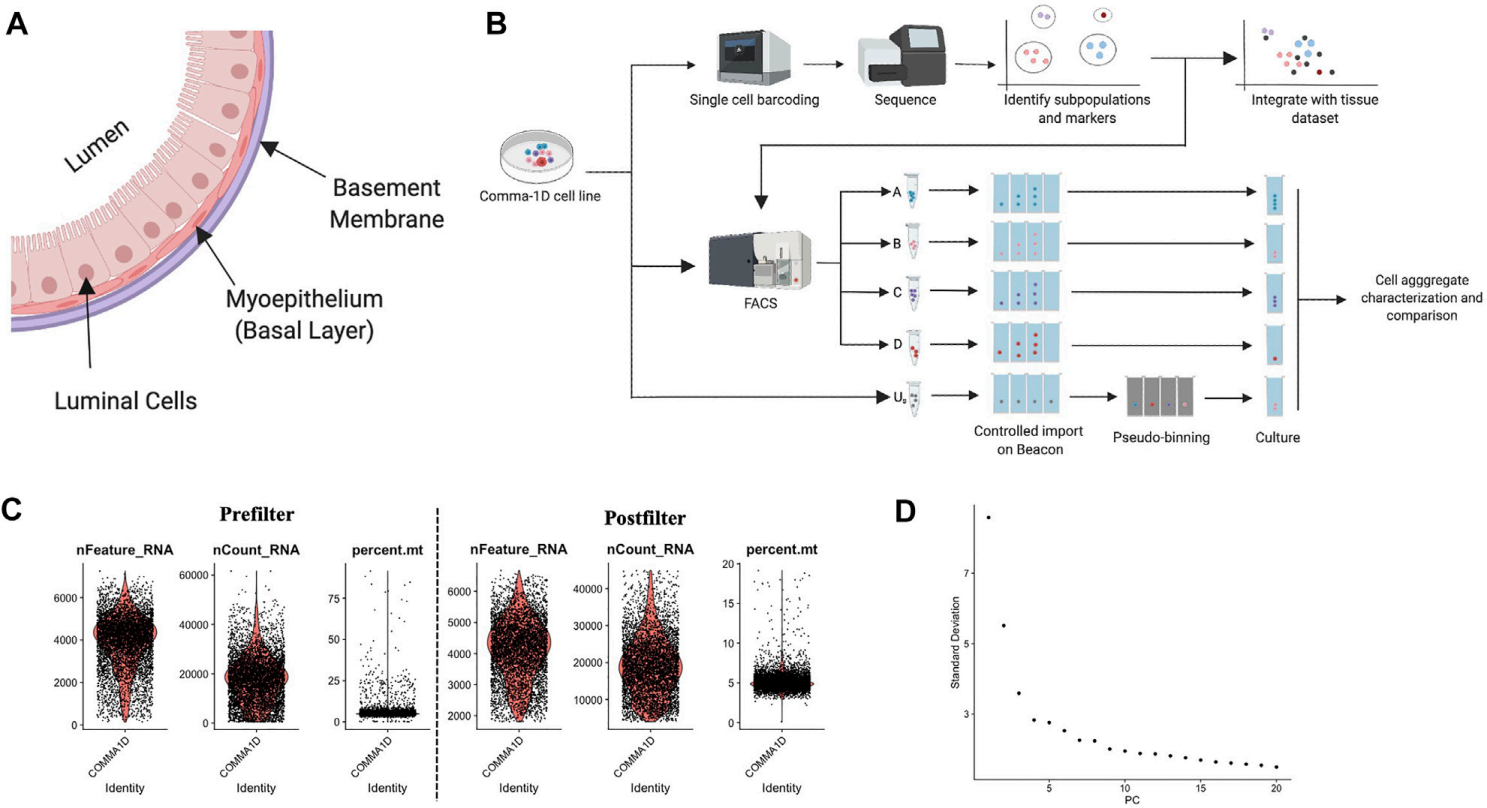
Background on mammary duct biology and an introduction of scRNASeq pipeline. **(A)** Model of mammary duct cross-section and potential cell types, including tumor initiation. **(B)** A framework of the experimental pipeline to investigate sample heterogeneity with improved resolution and throughput. The illustrations were generated on Biorender.com **(C)** Violin plots for percent of genes mapping to mitochondrial genome, the total number of molecules detected within a cell, and the number of unique genes detected in each cell. Each data point represents a unique barcode from a bead. Filtered violin plots for cells with the percent of genes mapping to mitochondrial genome less than 20%, the number of unique features detected in each cell greater than 2,000, and a nCount less than 45,000. **(D)** Elbow plot outlining the standard deviation/variance in the data set attributed to each principal component. Kinks in the curve can highlight the distinction between relevant and noise PCs.

In most mammalian females, the mammary gland consists of branching ducts surrounded by adipose tissue. The ducts comprise three layers: basement membrane, basal cells, and luminal cells outlined in Figure 1A (Malhotra et al., 2010; Kondov et al., 2018). The basal cell contracture assists milk transport through the ducts toward the skin surface. The luminal cells line the inside of the ducts, and the alveolar cell lineage secretes milk during pregnancy. Within normal development, the ducts branch throughout the breast, and adipose is the predominant tissue. Details of mammary gland development across developmental time points have been extensively studied (Hens and Wysolmerski, 2005; Anderson et al., 2007). Over the course of pregnancy, prolactin and progesterone trigger drastic branching and invasion of ducts along with lobuloalveolar units to maximize lactational competency. This tissue remodeling also involves environmental reorganization for supporting development, including vascularization to support growth, lipid loss in adipocytes for spatial restructuring, and enlargement of the liver for addressing increased energy needs (Petitti and Perlman, 1988). There is evidence highlighting the pathogenesis of breast cancer mirroring the functional pathways identified for mammary gland development during pregnancy. Mechanisms such as reduced cell apoptosis, increased cell proliferation, and extracellular matrix modification reflect alterations in oncogenesis and pregnancy (Slepicka et al., 2019). Therefore, model systems that can simulate properties specific to healthy mammary gland function have the potential to serve as a proxy for better understanding of breast cancer disease pathogenesis.

Characterization of breast cancer is often leveraged to classify patients into disease pathways based on surface receptor expression of Estrogen receptor (ER), Progesterone receptor (PR), and Human epidermal growth factor receptor 2 (HER2). As higher resolution assays reveal marker expression attributed to subpopulations within a tumor, the granularity of disease classifications has been modified to reflect this new information (Lehmann et al., 2016). Novel gene vectors within a population have also been shown to provide information on proliferative capacity, migration tendency, stemness capability, and treatment targets (Jiang et al., 2021; Xu et al., 2021). Further investigations into breast cancer have previously yielded fundamental marker discoveries including *Mki67* ^+^ as a prognostic marker, *Cd44* ^+^/*Cd24* ^+^ as a breast cancer stem cell marker, and *Trop2* as another therapeutic target (Ricardo et al., 2011; Xiong et al., 2019; Liu et al., 2021). Gene vectors also assist cell type deconvolution and functional prediction with markers such as epithelial cell adhesion molecule (*Epcam*), actin alpha 2 (*Acta2*), and collagen type 1 alpha two chain (*Col1a2*) expressed significantly in luminal, myoepithelial, and fibroblast cell types, respectively (Prater et al., 2014; Visvader and Stingl, 2014; Muhl et al., 2020). Epcam further serves as a marker for stemness and functions in cellular migration (Gaiser et al., 2012). Understanding the expression patterns of functional gene vectors in model systems ultimately allows for improved stratification of study systems and informed cell line selection in legacy investigations.

The Comma-1D cell line was derived from BALB/c mouse mammary epithelium and is known to functionally differentiate to preneoplastic and neoplastic phenotypes *in vitro* (Danielson et al., 1984). Cell lines traditionally serve as a high throughput model system to understand normal and oncogenic characteristics. Cell line models differ in their degree of homogeneity, with commonly investigated breast cancer lines such as MCF7 indicating biological differences between labs (Osborne et al., 1987). We chose to characterize Comma-1D using single-cell methods to underscore this model line for its known differentiation in culture as a demonstration to highlight the efficacy and resolution of this suite of methods. Moreover, the identification of subpopulations demonstrating stem-like gene expression followed by phenotypic assays to define differentiation capacity resulted in the development of multiple robust pipelines for model generation and characterization. The differential gene expression analysis (DGEA) analysis provided a framework to identify clusters based on transcriptomic and predicted genomic alterations. These clusters may have identifiable functional traits that can be observed and quantified. For example, cell motility is an increasing trait of interest in oncology (Twigger et al., 2015). With this pipeline, we can identify motile gene expression across the population through scRNASeq followed by high throughput single-cell fluorescence quantification of migratory proteins.

The advent of high throughput single-cell processing platforms has allowed for the deep characterization of known and novel cell subpopulations. Further, these assays have been employed to define tumor heterogeneity and investigate tumor microenvironment across disease subtypes and locations (Paul et al., 2017). However, single-cell technologies have not yet been leveraged to characterize many model systems, including Comma-1D, as many cell lines have been assumed to be homogenous and well defined based on findings from bulk sequence data. Consortiums such as The Cancer Genome Atlas provide conventional bulk RNA and DNA methylation for cell lines, which serve as a necessary foundation for any preliminary testing using these model systems (Ren et al., 2018). As our understanding of the complexity of cancer evolves, the resolution of data needed to provide an accurate framework for therapy targets needs to approach a single-cell level. By determining populations of functional variability, we generate a spectrum of subclonal populations, which can be ordered by predicted role in disease progression, role in tumor population heterogeneity, and downstream effect by successful therapeutic targeting. We present here an optimized high throughput method for single-cell genomic analysis for population identification to inform downstream phenotypic and functional experimentation (Figure 1B **)**. This method identified four novel populations of interest and enabled an 80.4% increase in microfluidic cell cultures populated with the *Epcam* ^*+*^ *Cd49f* ^*high*^ *Sca-1* ^*high*^, stem-like cell type.

## Materials and Methods

### Comma-1D Mouse Mammary Epithelial Culture

The Comma-1D cell line was provided by the Gregory Hannon Laboratory (Cancer Research United Kingdom, Cambridge Institute). Aliquots were thawed and then cultured in CytoOne T25 flasks (US Scientific) with culture media composing DMEM/F-12 media (Thermo Fisher), 2% FBS (Sigma-Aldrich), 1% Pen-Strep (Gibco), 10 μg/ml Insulin (Sigma-Aldrich), and 5 ng/ml Epidermal Growth Factor (Thermo Fisher). Upon 80% confluency, the sample was passaged following the recommended subculturing protocol for adherent cells (Cancer Genome Atlas Research et al., 2013).

### Single-Cell RNA Sequencing and Library Prep

For single-cell RNA sequencing, cells were collected at passage five and suspended in 1x PBS media at 1 × 10^6^ cells/ml. Cells were processed according to Chromium 3' Gene Expression V3 Kit (10X Genomics) using the manufacturer’s guidelines followed by sequencing on an S1 NovaSeq chip (Illumina Inc.). Qubit 3 (Fisher Scientific) and 2100 Bioanalyzer (Agilent) were used for quality check of cDNA. The output BAM file from sequencing was processed through 10X Genomics Cell Ranger software v3.1.0. The outputted read counts matrix inputs into R for downstream analysis such as Seurat and Monocle.

Single-cell gene expression data resolves unique heterogeneity information not attainable from conventional bulk sequencing technologies (Ricardo and Phelan, 2008). To evaluate the heterogeneity in the Comma-1D cell line, we ran the cells through the pipeline and successfully generated scRNASeq data with ∼50,000 2 × 150 bp reads per cell with 5,745 cells sequenced, generating 238 M reads with 98% valid barcodes and 100% valid UMIs. 85.3% of the total reads mapped to the mm10-3.0.0 reference genome.

### ScRNASeq Comma-1D Data Filtering and Analysis

To advance computational accuracy and remove predicted outlier data, the Seurat object representing the cells from the Comma-1D line was filtered for features that were not present in at least 15 cells (∼0.3% of total cell count) (Zheng et al., 2017). To further clean the data set, data points were filtered using three parameters: high percent mitochondrial data is indicative of cell death, therefore cells with >20% were removed; a high count of unique features > 45,000 are indicative of multiplets and ribosomal RNA (rRNA) in the gel emulsion (GEM) formation and were therefore removed; a low count of unique features <2000 is also indicative of GEMs with no cells or debris, and these cells were also removed from the dataset (Svensson et al., 2017; Butler et al., 2018; Freytag et al., 1000). Figure 1C represents the data pre- and post-sub-setting for the above-mentioned quality standards, respectively, with 5,195 of the 5,745 sequenced cells passing these QC parameters. After passing the initial QC, the data was normalized to account for variability. Cell cycle scoring was done to mitigate cell cycle heterogeneity through phase scoring of G2/M and S markers, which were then regressed out (Kimmerling et al., 2018). PCA was conducted for dimensionality reduction to identify undefined components that constitute variability within the data, plotted in Figure 1D. The PCA scores were used by Seurat to generate unsupervised clusters. The Seurat object with filtered and labeled data was then utilized by the Monocle pipeline for pseudo-time analysis. Data is imported through Monocle through extracting and expression matrix, cell metadata, and gene annotations from the Seurat object. The new cell data set is reprocessed and standardized through the preprocess_cds() function. The cell dataset it reclustered with the louvain_iter set to one, nearest neighbor k value set to 150, and a UMAP-based dimensionality reduction. Top genes from the Seurat clusters are leveraged as markers to annotate the Monocle generate populations.

### Integration of Comma-1D and Mouse Mammary Dataset

To better understand the functional populations predicted in Comma-1D, the dataset was integrated with previously published scRNASeq mouse mammary data from a C57BL/6 mouse (Pal et al., 2021). C57BL/6 is a widely used strain commonly utilized for developmental biology and therefore served as a good selection for comparison with Comma-1D. For functional comparison, the adult C57BL/6 mammary tissue dataset was downloaded from the GEO database (GSE164307) and integrated with the Comma-1D scRNASeq data. The dataset was imported and preprocessed with the same pipeline described for Comma-1D. Cells were filtered for identifiers with over 800 features, less than 20,000 total counts, and less than 20 percent mitochondrial gene activity. This dataset was independently normalized and scaled prior to data integration. The C57BL/6 mouse mammary dataset provided 11,997 total cells post-filtering. Harmony is a data integration algorithm that encourages cell grouping by cell type rather than dataset biased metrics by accounting for experimental variability and was used for the integration of these data (Korsunsky et al., 2019). As done for the Comma-1D dataset independently, the Harmony merged dataset was processed for global unsupervised clustering using FindNeighbors() and FindClusters(), with a resolution value of 0.5. Clusters were analyzed using the FindAllMarkers() function and gene markers used to identify partitioning Comma-1D were replotted for the merged dataset using feature plots.

### Ranked Stemness Prediction Using Entropy Scoring of Comma-1D Clusters

To estimate the differentiation potency (i.e., stemness) of single cells, we leveraged a computationally accelerated calculation of transcriptional Shannon entropy, following work pioneered by Tessechndorf and Enver (Teschendorff and Enver, 2017). Conceptually, differentiation of a cell involves the progressive silencing of gene pathways unrelated to its increasingly specialized function, such that the promiscuity seen in transcriptional activity decreases as a cell differentiates. Conversely, stem-like cells retain a higher degree of overall transcriptional activity across a multitude of pathways governing potential fates. This dynamic can be leveraged to estimate the degree of differentiation of a single cell, through calculating the Shannon entropy rate of a random-walk across a protein-protein interaction (PPI) network with gene expression superimposed.

We implemented entropy estimation using the abovementioned approach in Tensorflow 2.0 with support for GPU accelerated calculation. Prior to entropy scoring, unnormalized raw cell counts were subject to light kNN smoothing (*k* = 8) to reduce technical variance in entropy scores induced by gene dropout and subsequently renormalized to 10,000 counts per cell. Entropy scoring on smoothed, depth-normalized count data was performed on a Google Cloud VM n1-highmem-16 instance running on an NVIDIA Tesla T4 GPU with 16 GB RAM. After calculating entropy scores, unsmoothed, depth-normalized counts were used to calculate spearman correlations for all genes with respect to entropy score. Significance values were adjusted to account for multiple testing correction using the benjamini-hochberg false discovery rate method. Genes with positive spearman correlation are overexpressed in stem-like cells, while genes with negative spearman correlation are overexpressed in differentiated cells.

### Subpopulation Tracking and Stemness Evaluation With Beacon

Cells were harvested from culture at passage five and suspended in Comma-1D culture media at an ideal loading concentration of 2 × 10^6^ cells/ml. Utilizing the small volume import parameters on Beacon, 5 µl of media with cells were bubble imported into the microfluidic chip at 25°C. OptoElectroPositioning (OEP) was used to identify, isolate, and pen both independent cells and clusters of cells into isolated nanopens. Cells were loaded with a voltage of 2.1 V at 5 μm/s, with a target of 1–4 cells per pen. This import and culture method has previously been utilized for cellular characterization (Beaumont et al., 2022). Over multiple iterations of this importing and culture, levels of Matrigel (Corning) were optimized to allow cell adherence on the chip surface while also providing an isolated nanopen network for 3-dimensional growth. The cells are cultured on chip with DMEM/F-12 media (Thermo Fisher), 2% FBS (Sigma-Aldrich), 1% Pen-Strep (Gibco), 10 μg/ml Insulin (Sigma-Aldrich), and 5 ng/ml Endothelial Growth Factor (Thermo Fisher) at 37°C with 5% CO_2_. The microfluidics on-chip allow constant perfusion of media which is perfused at 0.01 μl/s. Fresh media refills is provided to the system every 24 h. Cells were cultured up to 10 days post-import.

All nanopens and their cellular contents were imaged in 12-h and 24-h intervals comprising every imaging channel on the Beacon system (OEP, DAPI, FITC, Texas Red, or Cy5). Pseudobinning was performed with differential expression of fluorescence of antibodies EpCAM-Cy5 (Biolegend #118220), EpCAM-AF594 (Cell Signaling Technology #73195), CD49f-FITC (Biolegend #313606), and Sca-1-BV421 (Biolegend #108127). The images were then collated and analyzed on a custom MATLAB (MathWorks) script. Images captured across timepoints and channels are aligned through the microfluidic chip’s feature points. Each nano pen is designed with a region of interest (ROI) for coordinate-based image comparison between timepoints and imaging channels. Cells are identified through a circle detection algorithm that then filters possible cell locations by image intensity readings under the brightfield (OEP) channel. True positive cell locations are saved on a 3-dimensional matrix of coordinates. Each ROI and each cell location are measured for fluorescent intensity across channels at t_0_. These values are normalized by dividing cell location measurements by that cell’s ROI measurement. This normalization permits multi-chip comparison and scoring. The normalized image intensity readings are clustered based on populational heterogeneity identified from single-cell sequencing data. The cell intensity readout is binned into the generated clusters allowing for high throughput post-import cellular subtype identification.

## Results

### Resolving Subpopulation Heterogeneity Within Comma-1D With Single-Cell Transcriptomics

**FIGURE 2:**
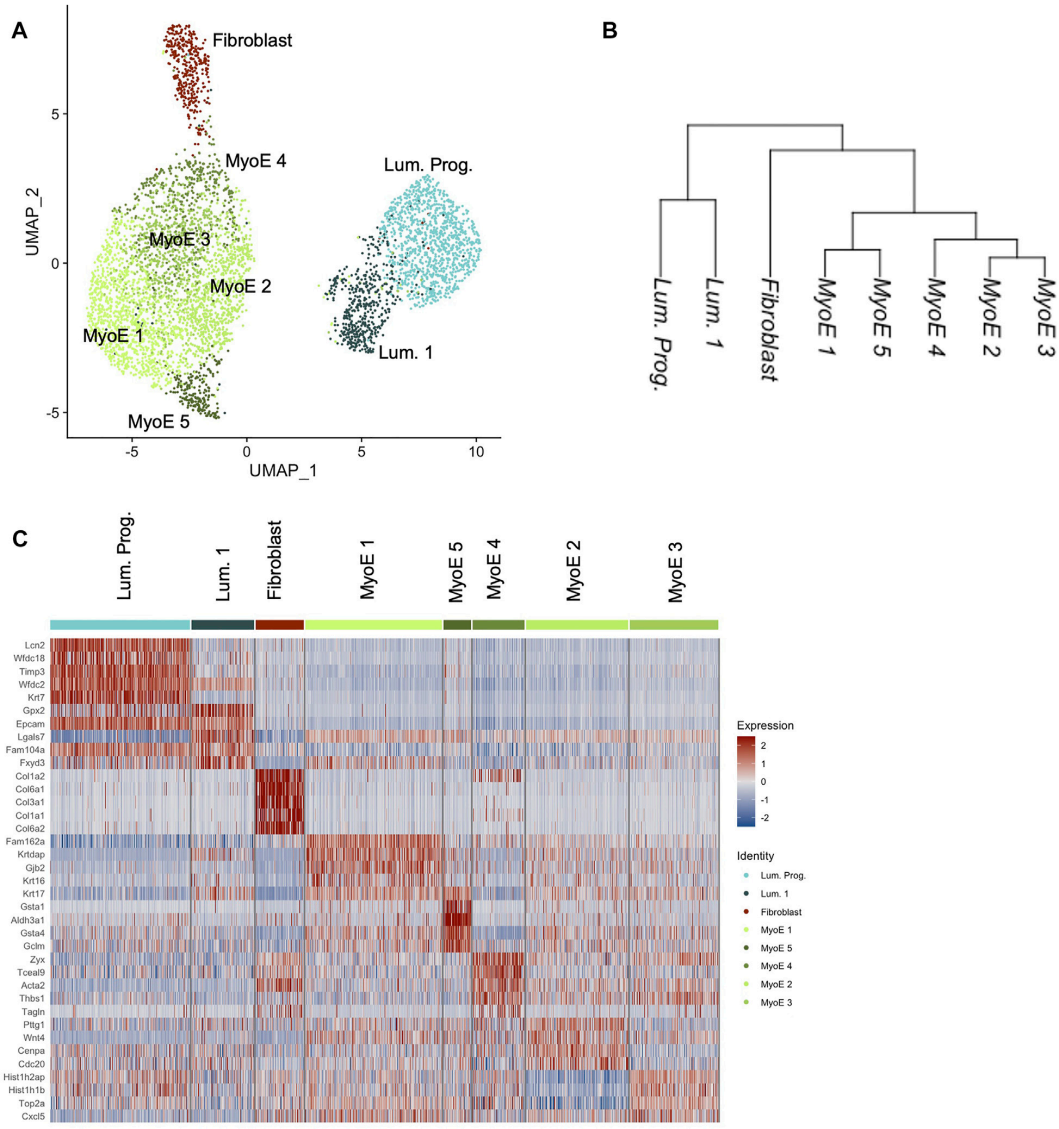
Comma-1D preliminary cluster analysis. **(A)** The UMAP depicts unsupervised clusters across the population with a resolution parameter of 0.5. **(B)** Cluster tree generated by averaging data points across a cluster and deriving distance relationships between the identified populations on a pseudo-bulk level. **(C)** The heatmap outlines the expression of the top five DEGs within each cluster if they pass the significance threshold.

We generated eight unique subpopulations from unsupervised clustering of our Comma-1D cell line culture sample. Principal Component Analysis (PCA) is useful for fast and linear dimensionality reduction, however with increasingly complex data affiliated with scRNASeq, UMAP is another preferred network analysis tool that preserves the global structure, distance correlations, and continuity of cell states (Xiang et al., 2021). Figure 2A shows a UMAP plot of the heterogeneity of the evident subgroups. Using the filtered, normalized, and scaled dataset, cell line clustering provided an increased resolution to gene expression and clonal population differences. Investigating differential gene expression between clusters within this dataset, we further highlight potential functional clustering. While one dataset was generated and processed for scRNASeq of the Comma-1D cell line, the throughput of 5,195 cells provides confidence in characterizing the observed heterogeneity. Datasets were generated for functional validation using Beacon data across 1,214 cells from two individual chips to demonstrate technical validity. All *p*-values are reported for each differentially expressed gene (DEG) analysis; however, due to the throughput of this assay and a high n count, *p*-values are close to 0 with values less than 2.225074e-308 reported as 0 by R. Figure 2B and Supplementary Figure S1 are cluster trees outlining distance relationships between the generated subgroups, with Supplementary Figure S1 indicating nodal identifiers for downstream analysis and reference. This phylogenetic tree analysis averages data points across an identified subpopulation to extrapolate distance relationships between the identified clusters. Top differentially expressed genes driving the node splits in the population are outlined in Supplementary Table S1. These gene vectors generate hits relevant to interpreting functional identities of these populations, such as *Lcn2* (*p* = 0) and *Col6a1* (*p* = 0). From the cluster tree and UMAP in Figure 2, we visualize specific populations as more distant from the remaining cells, primarily clusters attributed to fibroblasts. To investigate the functional hallmarks of these populations, we explored all markers expressed in each cluster and sorted them by the difference of pct.1 and pct.2, which represent the percent of cells in a specific cluster expressing a gene and the percent of cells outside that cluster expressing that gene, respectively. Typically, we have identified that genes with difference values greater than 0.5 are responsible for providing the most direct representation of individual cluster states and that these genes, in most cases, parallel the most significant DEGs with regards to *p*-value and avg_logFC. Through sorting by this difference value, we derive gene expression sets increasingly specific to the subpopulation of interest (Newman et al., 2015). This analysis was run across all the generated clusters; resultant data is shown in Supplementary Figure S2.

**FIGURE 3:**
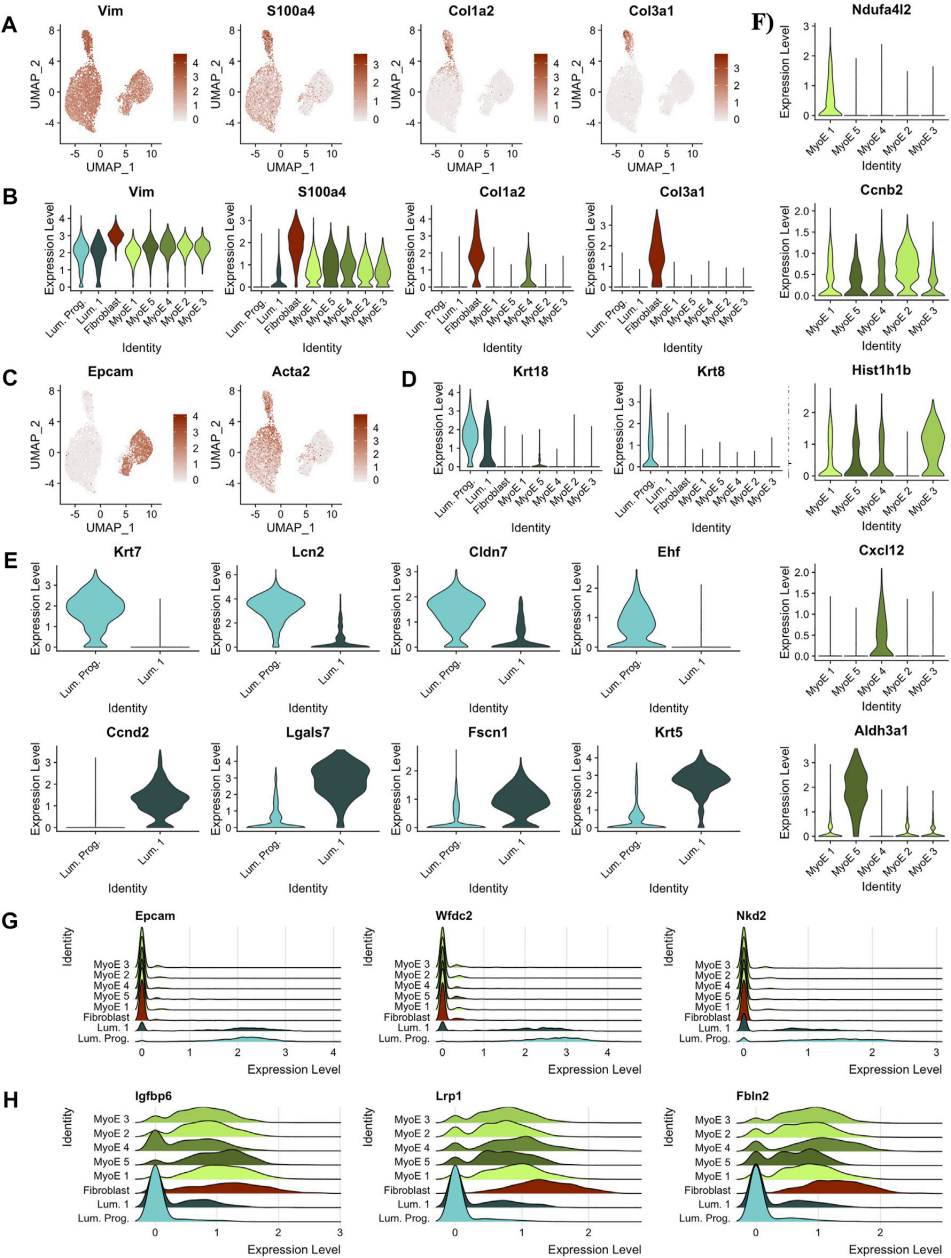
Predicting cell types and functions by cluster. **(A)** Feature plots of key canonical markers of fibroblastic cells **(B)** Violin plots of key canonical markers of fibroblastic cells. Identified fibroblast cluster exclusively expressed all markers. **(C)** Feature plots depicting expression of markers derivative of luminal and myoepithelial subtypes, EPCAM and ACTA2, respectively. **(D)** Violin plots outlining distinct expression of Krt18 expression within the luminal subtypes indicating possible alveolar cell function. Krt8 expression was significantly isolated to the predicted luminal progenitor population within the luminal subtype. Almost exclusive expression of Krt8 indicates luminal progenitor cluster as a progenitor population within luminal cells and with luminal 1 as slightly differentiated cells with secretory/alveolar function. **(E)** Differential genes between the two clusters in the luminal partition. **(F)** Differential genes within the local myoepithelial partition. **(G)** Ridge plot of gene markers conserved within luminal cells, but not expressed significantly outside the luminal population. **(H)** Ridge plot of gene markers conserved within myoepithelial cells, but not expressed significantly outside the myoepithelial population.

The top five differentially expressed genes per cluster that pass a baseline significance criterion are shown on the heatmap in Figure 2C. These top differentially expressed genes were parsed to identify the potential cell types as labeled. The top five genes in the cluster predicted as fibroblast are *Col6a1* (*p* = 0), *Col3a1* (*p* = 0), *Col6a2* (*p* = 0), *Gng11* (*p* = 0), and *Pdgfrb* (*p* = 0). These genes highlight invasion in the Comma-1D population as part of the diversity of collagen genes typical of fibroblast cells (Olsen et al., 1989). Previous studies have also found fibroblasts in Comma-1D cell line culture (Danielson et al., 1984). Further evidence supporting the presence of a fibroblast population in Comma-1D is highlighted in Figure 3A by the feature plots generated across typical canonical markers of fibroblastic cells. The fibroblast cluster is significant in its isolated expression of *Col3a1* (*p* = 0), while also expressing the *Vimentin* (*Vim*, *p* = 1.34e-154), *S100a4* (*p* = 3.06e-164), and *Cola2* (*p* = 0) at a higher intensity than the remaining populations. Violin plots shown in Figure 3B quantify the observed differential expression amongst the genes highlighted above.

This pipeline was applied in parallel to the additional clusters identified in the Comma-1D culture. Through these analyses, we identified the remaining functional groups composing the Comma-1D culture population. Twigger et al. (2015) identified key gene markers that functionally determine mammary gland cell populations, including two main types of epithelial cells, determined to be luminal and myoepithelial cells. Figure 3C shows increased gene expression of *Epcam* (*p* = 0) and *Acta2* (*p* = 0) used to identify luminal and myoepithelial populations, respectively. Diving deeper into each predicted partition, within the luminal subgroup, we observed a higher-than-expected expression of *Krt18* (*p* = 1.57e-79), a marker for the alveolar subtype of luminal mammary cells Figure 3D (Moritani et al., 2015; Zhao et al., 2010). Much like Zhao et al. (2010) noted in their immortalized mammary stem/progenitor cells, we see the expression of *Krt8* (*p* = 0) isolated to the luminal progenitor cells relative to all other cell populations, shown in Figure 3D (Lichtner et al., 1991). After running a FindMarkers() function, we generate genes differentially expressed between the luminal progenitor population and luminal 1 population, with the top hits sorted by pct.difference visualized by the violin plots in Figure 3E. From the DEGs identified in Figure 3E, we noticed that the *Krt7* (*p* = 1.13e-180) and *Ehf* (*p* = 1.78e-68) expression, known to identify immature luminal epithelial cells, was isolated to the luminal progenitor cluster (Jones et al., 2004; Kumar et al., 2018).

Within the breast, the luminal progenitor cells differentiate into non-secretory epithelial and alveolar cell types for lactating function (Booth et al., 2007). The non-secretory epithelial cells are typically identified by *Krt19*, for which we did not observe expression within this population in our experiments. However, we did observe *Krt18* expression, associated with the milk-creating alveolar subtype, in the luminal progenitor cluster, as shown previously in Figure 3D. The Violin plots in Figure 3F identify a significant gene within each cluster compared to the local myoepithelial population. We found identifying functional differences within the myoepithelial clusters to be too assumptive based on our scRNASeq data; however, some DEGs can provide useful inferences. For example, the myoepithelial 5 population significantly expressed *Aldh3a1*(*p* = 1.48e-157), which has been linked with increased cell proliferation and tolerance to the cytostatic and cytotoxic effects of lipidic aldehydes (Muzio et al., 2012). We also identified the differential genes across all the clusters within the myoepithelial cell type in the heatmap shown in Supplementary Figure S2A. To highlight the differential expression for key markers, feature plots for a top gene per cluster is visualized in Supplementary Figure S2B. This differential gene expression analysis can be paralleled across any two cell selections within the population.

Running a differential gene expression analysis between the luminal and myoepithelial partitions generates gene vectors conserved within each partition but with differential expression between the populations. Table 1 lists the top genes that were differentially expressed between myoepithelial and luminal subtypes, identified by selecting the top 10 genes by the difference in the percentage of cells in each partition expression that marker. The top three DEGs are visualized on the ridge plots in Figure 3G showcases genes conserved within luminal cell types while also demonstrating a lack of expression in the myoepithelial cells, such as *Epcam* (*p* = 0), *Wfdc2* (*p* = 0), and *Nkd2* (*p* = 0). Similarly, Figure 3H represents the top three genes conserved within myoepithelial cell types with a lack of expression in the luminal cells, such as *Igfbp6* (*p* = 0), *Lrp1* (*p* = 0), *Fbln2* (*p* = 0).

**TABLE 1:** Top genes differentially expressed between luminal and myoepithelial subtypes. Conserved genes in the myoepithelial and luminal partitions that are differentially expressed between the two. *p*-Value less than 2.23e-308 is reported as 0 by R.

**Luminal conserved gene markers**			
**Gene**	**Percent expression in luminal population**	**Percent expression in myoepithelial population**	***p*-value**
Epcam	0.917	0.066	0.000000e+00
Wfdc2	0.923	0.11	0.000000e+00
Nkd2	0.816	0.061	0.000000e+00
Krt18	0.848	0.11	0.000000e+00
Lsr	0.749	0.021	0.000000e+00
Cldn7	0.733	0.016	0.000000e+00
Lad1	0.789	0.079	0.000000e+00
Rab25	0.671	0.017	0.000000e+00
Wfdc18	0.72	0.082	0.000000e+00
Krt7	0.669	0.054	0.000000e+00
**Myoepithelial conserved gene markers**			
**Gene**	**Percent expression in luminal population**	**Percent expression in myoepithelial population**	***p*-value**
Igfbp6	0.193	0.888	0.000000e+00
Lrp1	0.208	0.885	0.000000e+00
Fbln2	0.223	0.882	0.000000e+00
Ass1	0.246	0.895	0.000000e+00
Sparc	0.371	0.999	0.000000e+00
Ptges	0.157	0.776	0.000000e+00
Igfbp2	0.149	0.766	0.000000e+00
Pdpn	0.186	0.802	0.000000e+00
Emp3	0.372	0.985	0.000000e+00
Ly6e	0.364	0.976	0.000000e+00

Each subcluster identified has unique signatures that assist in defining its local and global functionality within a heterogeneous cell line. To assist in functional predictions, top gene vectors for each subcluster, identified by significant avg_logFC value, are ported through a gseGO network analysis provided by the ClusterProfiler() package (Wu et al., 2021). The gene vectors are generated to compare functionality within each partition rather than global comparison, with the exception of the predicted fibroblast population. For example, the gene vector used to run the network analysis for myoepithelial 1 was generated by comparing the gene expression of that cluster to the remaining myoepithelial subclusters: myoepithelial 2, myoepithelial 3, myoepithelial 4, myoepithelial 5. These local comparisons within each partition determine differential transcripts with functional indications. All the pathway predictors and the affiliated genes for each subpopulation are visualized by the Cnet plots in Supplementary Figure S3. For each subcluster analysis, the Cnet plots illustrate top pathway activation indicators and their respectively linked genes. The pathways identified predict functional differences in each subcluster based on known gene vectors. The myoepithelial subclusters were of interest in this analysis to parse functional granularity between clusters. Myoepithelial 1 expressed genes associated with cellular response to external stimuli, like *Mt1* (*p* = 1.07e-100), *Mt2* (*p* = 1.78e-179), and *Bnip3* (*p* = 8.76e-150). The expression of *Pttg1* (*p* = 4.95e-120) and *Cdc20* (*p* = 1.35e-53) in myoepithelial 2 indicated pathway activation related to reproduction and cell growth. Myoepithelial 3 was defined by genes relating to gene ontology (GO) terms for cellular components and anatomy, like *Cdk1* (*p* = 3.65e-49) and *Zyx* (*p* = 8.46e-88). The top DEGs in myoepithelial 4 were associated with GO terms for response to wound healing and cell proliferation, including genes like *Tpm1* (*p* = 2.15e-41), *Fn1* (*p* = 4.31e-71), and *Sparc* (*p* = 2.62e-100). The last population of myoepithelial cells, myoepithelial 5 was distinguished by the nucleus and intracellular organelle lumen pathways, including genes such as *Hsph1* (*p* = 9.93e-22), *Cbr3* (*p* = 4.57e-68), *Ptges* (*p* = 1.48e-41), and *Aldh2* (*p* = 2.78e-33). The myoepithelial 5 population pathways have high gene counts therefore only two pathways are visualized, whereas in the other plots top three are shown. Luminal 1 has gene enrichment of *Krt17* (*p* = 1.31e-164), *Krtdap* (*p* = 3.06e-216), and *Krt6a* (*p* = 2.70e-242) linked to pathways involved in keratinization and epidermis development. The luminal progenitor population has pathway activation of protease binding and endopeptidase inhibition, indicated by the expression of genes such as *Wfdc18* (*p* = 0) and *Lcn2* (*p* = 0). The fibroblast population has activation of genes related to extracellular matrix (ECM) and external structure activation through gene expression of *Col1a1* (*p* = 0), *Col1a2* (*p* = 0), *Col3a1* (*p* = 0), and *Col6a1* (*p* = 0). The significance of expression is lower among the myoepithelial comparisons further indicating that the population has reduced functional heterogeneity in comparison to the two luminal clusters.

### Subpopulation Comparison of Integrated C57BL/6 Mouse Mammary and Comma-1D Data

Harmony was used to compare populations between cell line and mouse mammary tissue. The merged dataset was re-normalized to reduce experimental and sample bias. The UMAP in Supplementary Figure S4A illustrates the relationship proximity between sample types. The merged dataset was processed for unsupervised clustering which yielded ten unique subpopulations composing luminal, myoepithelial, and fibroblast partitions are shown in Supplementary Figure S4A. The bar plot in Supplementary Figure S4B quantifies sample contribution to each cluster. Every cluster population contains cells from both samples; however, most clusters have a preferential population to a given sample. For example, the myoepithelial 1 cluster has 1,944 total cells with 362 cells (18.62%) originating from C57BL/6 and 1,582 cells (81.38%) from Comma-1D. The populational breakdown linked with cell locations on UMAP indicates similar functional populations representative in each sample type, illustrated in Supplementary Figure S4C. The three feature plots summarize the expression of canonical markers *Col1a2*, *Acta2*, and *Epcam* to identify fibroblast, myoepithelial, and luminal cell types, respectively. These partitions of cells are detected across the Comma-1D and C57BL/6 datasets. From the heatmap in Supplementary Figure S4D, top differential genes characterizing each cluster is visualized. The merged analysis revealed two smooth muscle cell derived myoepithelial populations, indicated by the expression of canonical markers *Tagln* (*p* = 0) and *Tpm2* (*p* = 0). One of the luminal populations indicated ductal cell functionality with the expression of markers *Csn3* (*p* = 0) and *Wfdc18* (*p* = 0). This ductal specific luminal population was not previously identified by the Comma-1D independent analysis.

### Resolving Founder Populations Through Pseudo-Time Analysis

**FIGURE 4:**
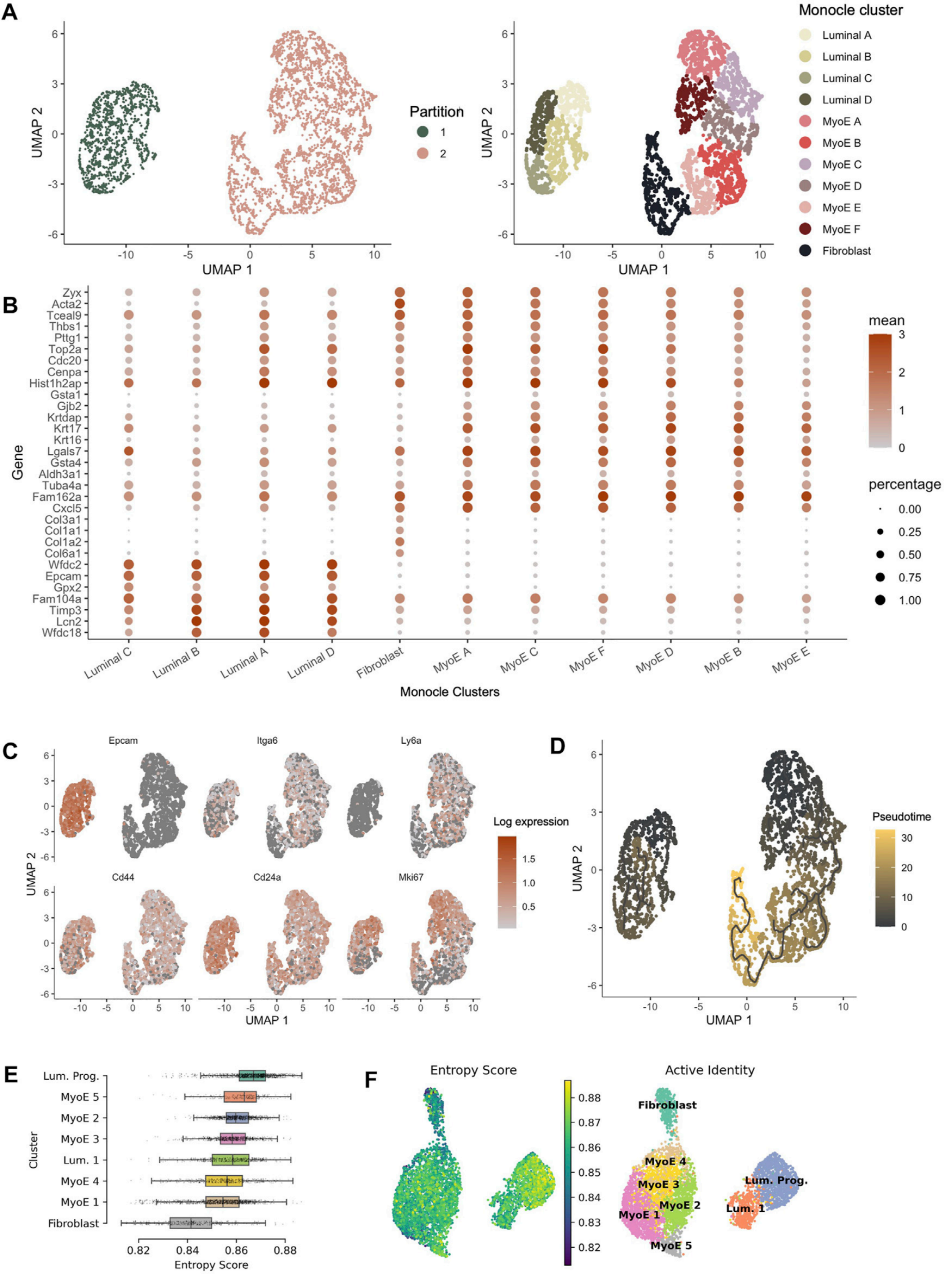
Pseudotime analysis of Comma-1D populations. **(A)** Monocle partitions and reclustering within the populations. Monocle clustering generated 11 total clusters composed of luminal, myoepithelial, and fibroblast populations. **(B)** Dotplot of the top four genes per cluster identified from the Seurat generated Comma-1D populations. Gene expression is plotted against Monocle generated clusters **(C)** Feature plots illustrating expression of cancer stem cell (CSC) markers *Epcam*, *Itga6*, *Ly6a*, *Cd44*, *Cd24a*, and *Mki67*. Significant stemness related gene markers observed in the luminal A and myoepithelial A Monocle clusters. **(D)** Utilizing manually selected root nodes of interest, we create a trajectory predicting population evolution. Within Partition 1, luminal C is furthest along the differentiation trajectory. The fibroblast population is most progressed along the differentiation trajectory in Partition 2 **(E)** Ranked entropy score by Comma-1D cluster, luminal progenitor population with most stem-like capability in this cell line. **(F)** UMAP plot overlaid with entropy score gradient for Seurat generated clusters.

The Seurat package provides a comprehensive pipeline for cluster identification and differential gene expression. As single-cell data increasingly becomes a more widespread tool for advancing the efforts of onco-genomics, more analysis platforms are providing analysis tools to parse and interpret this complex landscape. For example, the Trapnell lab has generated the Monocle 3 pipeline for pseudo-time analysis across a population at a given time point (Trapnell et al., 2014). Using the differential gene expression across an entire population, we can use Monocle 3 to visualize predicted nodes of origin and differentiation. We see parallel functional clustering when investigating our COMMA-1D cell line through both pipelines. Monocle reclustering generated 11 unique populations within Comma-1D. The clusters generated resemble the luminal, myoepithelial, and fibroblast transcriptomic profiles, identified previously using Seurat, outlined in Figure 4A. The plot in Figure 4B illustrates top gene markers from the Seurat clusters and their expression profiles within the Monocle generated populations. We identified gene expression of *Col3a1* (*p* = 0), *Epcam* (*p* = 0), and *Acta2* (*p* = 2.37e-273), as predictive markers for fibroblast, luminal, and myoepithelial cells, respectively. As highlighted in Figure 4B, the representative gene expression patterns were observed across the various partitions of Comma-1D. Therefore, we can conclude the populations created through Monocle are separating cell types similar to cell groups we defined through Seurat cluster analysis, providing independent and orthogonal validation of our approach. We can apply this association to identify functionally similar populations between the analysis pipelines. For example, *Lgals7* is a gene marker for luminal 1 (*p* = 1.89e-109) in Seurat with differential expression in the luminal C (*p* = 3.48e-12) cluster in Monocle. Similarly, *Lcn2* is differentially expressed in the luminal progenitor (*p* = 0) cluster from Seurat with significant expression observed in luminal A (*p* = 5.88e-139) in Monocle.

Monocle pseudotime trajectories determine gene expression changes across the populations to place single cells along that defined trajectory. Top cancer stem cell (CSC) markers leveraged in Seurat are visualized on the Monocle UMAP in Figure 4C. This reveals populations such as luminal A and myoepithelial A with high predicted stemness capacity. In contrast, luminal C and fibroblast clusters have reduced expression of these stemness markers. Using the DEGs between clusters identified in Seurat, we generated a Monocle trajectory plot for those genes to determine which nodes yield key expression differences. The identified and selected nodes are used to order and plot cells on a pseudotime trajectory shown in Figure 4D. Based on the pseudotime trajectory results, we identify luminal A and myoepithelial A as populations early in the differentiation trajectory. These pseudotime findings support assumptions made from CSC marker expression. The trajectory analysis indicates luminal C and fibroblast populations as furthest along the differentiation trajectory within each partition. Based on parallel gene expression markers between analysis pipelines, this supervised pseudotime analysis further supports predictions from gene expression about the luminal progenitor population’s stemness capability.

To validate the supervised findings from Monocle 3, we employed an unsupervised single-cell entropy scoring algorithm to estimate stemness within the Comma-1D clusters. Entropy scores have indicated a correlation to increased stemness capability (Teschendorff and Enver, 2017). This analysis confirmed the luminal progenitor cells have the highest mean entropy score, Figure 4E. Thereby, further indicating this cluster’s potential function as a progenitor or stem-like population within the Comma-1D cell line culture. Using this tool, we identify fibroblast cells as the population with the lowest mean entropy score. As this is the most functionally differentiated population in our dataset, the findings support both key conclusions interpreted from entropy scoring. The other six clusters represent similar entropy scores with mean scores between those generated for fibroblast and luminal progenitor clusters. Figure 4F is the UMAP plot structure for Comma-1D overlayed with an entropy score gradient for each cell point. The plot visualizes the increased entropy within the luminal progenitor population.

### Comparative Analysis of Novel and Known Stem-Like Markers Within Comma-1D Population

**FIGURE 5:**
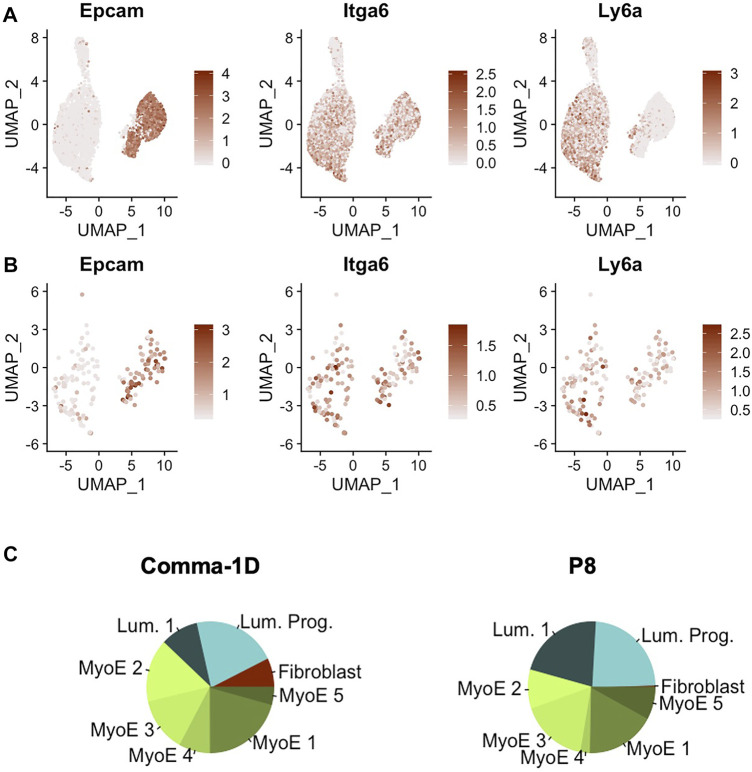
Gene expression of markers identified for stemness. **(A)** Feature plots of *Epcam*, *Itga6*, and *Ly6a*, which represents for the proteins *Epcam*, *Cd49f*, and *Sca-1* **(B)** Feature plot expression of the key gene markers after filtering for cells expressing a minimal threshold of 0.3 for all markers. **(C)** Pie plot visualizing population broken down by cluster. Between the entire Comma-1D population and P8, there is increased occupancy of luminal 1 and myoepithelial 4 with a significant decrease of myoepithelial two and loss of the fibroblast cell population.

Recent publications have highlighted the Comma-1D line for its inclusion of populations of stem-like cells (Yang et al., 2017). Parsing published data for markers of stem-like cells revealed three markers of stem-like cell subpopulations, *Epcam* ^*+*^ *Cd49f* ^*high*^ *Sca-1* ^*high*^, as well as preliminary phenotypic data supporting differentiation capability to both basal and luminal lineages (Krebsbach and Villa-Diaz, 2017). In addition, the expression of these markers has already been associated with disease prognosis (Yang et al., 2012). When searching for these markers across the cells sequenced from the Comma-1D line, we observed the differential gene expression patterns outlined in Figure 5A. The gene equivalent for the proteins *Epcam*, *Cd49f*, and *Sca-1* are the gene symbols *Epcam*, *Itga6*, and *Ly6a*, respectively. Combinatorial expression of these three markers yields unique cell selections identified by the nomenclature elucidated in Table 2. We subset the entire Comma-1D population for cells expressing one or more genes of interest to investigate the subpopulations further. For example, to isolate a P8 subpopulation, we filtered for cells with a scaled expression for the collective gene set, *Epcam* ^*+*^ *Itga6* ^*high*^ *Ly6a* ^*high*^, demonstrating higher than 0.3 intensity. This intensity cutoff was determined from a bimodality of expression across these genes where 0.3 was the lower bounds of the higher mode, which we believe correlates to true RNA expression, which can be visually interpreted by the violin plots in Supplementary Figure S5. Published markers *Cd44*, *Cd24*, and *Mki67* have each been linked with stemness potential and were plotted for comparison against *Epcam*, *Itga6*, and *Ly6a* expression (Ricardo et al., 2011; Cidado et al., 2016)*.* These P8 filtered cells were plotted in the feature plots shown in Figure 5B visualizing the stem gene markers of interest. The composition of this sub-selected dataset is outlined in the pie charts in Figure 5C, where we observed cells from all clusters in the original population present in this predicted stem-like population, except for the fibroblast cluster.

**TABLE 2:** Subpopulations of interest by identified marker expression patterns. Overview of markers selected for stemness investigation. Four subpopulations of interest were identified by expression patterns of epithelial cellular adhesion molecule, integrin subunit alpha 6, and lymphocyte antigen 6 complex locus A.

Subset	Epcam (Epcam)	Itga6 (Cd49f)	Ly6a (Sca-1)
P5	−	High	Low
P6	−	Low	Low
P7	+	High	Low
P8	+	High	High

### Custom Beacon Pipeline for Resolving Phenotypic Heterogeneity and Cell Aggregate Development

**FIGURE 6:**
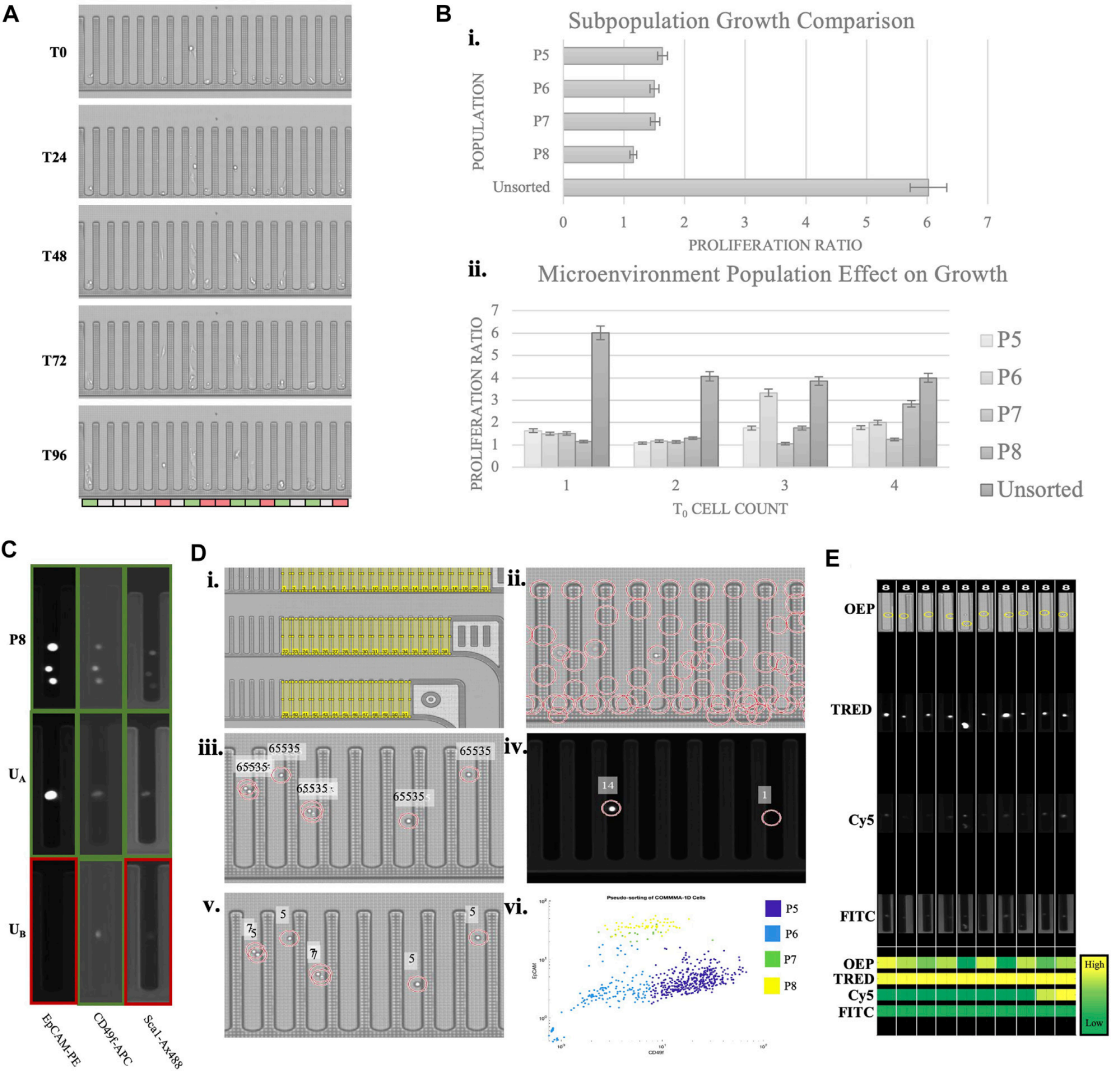
Phenotypic assay overview. **(A)** A subsection of a beacon chip across five time points of a culture period. The bar on the bottom indicates heterogeneity in observed growth response. Red indicates dying cells, white is no significant observed response, and green is for cells with detected proliferation **(B)** The top graph is plotting the growth ratio across identified subpopulations. The bottom graph indicates growth differences depending on initial cell concentration. 5% error bars are visualized for each plot. **(C)** Sample pen images of various cells across imaging channels. The colored outline is to highlight expression (green) or lack of (red). The top row is a known P8 population imaged at three channels with expression detected for all three markers. U_A_ and U_B_ indicate sample images of unknown cell types with marker expression pattern resolving subtypes P8 and P5, respectively **(D)** Overview of MATLAB script pipeline to identify cell populations by protein expression, automating observations described in **(C)**. **(Di)** Illustrates regions of interest (ROIs) representing each nanopen. **(Dii)** Shows possible cell detection locations based on circle detection. **(Diii)** Visualizes filtered cell detections leveraging OEP fluorescence thresholds. Fluorescence intensity above 65,535 is set as the threshold for true positive cell detection. **(Div)** Visualizes normalized expression scores for two cell detection locations in the TRED channel. Normalized scoring accounts for variability in background fluorescence between FOVs and between experimental chips. Readings are imported into a matrix for each detected cell for each marker and channel. **(Dv)**A sample readout of cells subtype based on the measured expression. Numbers “5” and “7” indicate populations P5 and P7, respectively. **(Dvi)**A scatter plot of all the detected cells plotted based on their expression of Cd49f and Epcam and the subtype they are predicted to belong to. 88.3% of FACS sorted cells pseudo-bin to same population on-chip. Each color represents the pseudo-binned subtype for that cell, as indicated in the legend. Nanopens with multiple cells at t_0_ are sources of noise and background fluorescence that misidentify some cells. **(E)** ROI images of randomly selected P8 identified cells from each imaging channel. The heatmap on the bottom compares expression across channels and cells. This functionality provides verification to the pseudo binning results, allows for clustering and threshold adjustment, and provides easy visualization for every cell subpopulation of interest.

The data presented was collected using a microfluidic instrument that allows high throughput cellular selection and manipulation with light-induced dielectrophoresis (Berkley Lights, Inc.). The system employs disposable microfluidic chips with 3,500 nanopens allowing for isolated cell cultures. Above the experimental chip is an integrated fluorescent microscope that operates with OEP, DAPI, FITC, Texas Red, and Cy5 channels. Using this system, experimental assays are designed to quantify variables including sample heterogeneity, response to stimulus, and cell interactions. Experimental and cultural conditions need to be optimized for each sample to induce assay robustness. Incremental adjustment of Matrigel concentration, cell import concentration, and chip surface treatment were done to improve cell adhesion, increase cell growth, and reduce processing time. Through dozens of iterations, we have identified a reproducible custom protocol, which resulted in Comma-1D cell growth to cell aggregates on the Beacon platform. Figure 6A demonstrates the Comma-1D cell line cultured under a controlled microenvironment over a 5-day culture period. Each image represents a progressive time with 24-h interval image capture of a fixed subsection of 18 nanopens on the instrument’s microfluidic chip. We observed cell-dependent growth differences between isolated cultures, resulting in the development of a method for capturing the biological differences driving cell-to-cell growth rate differences. The colored bar below pen images indicates pens with cells that either proliferated (indicated by green) or showed signs of apoptosis (indicated by red).

We performed a growth analysis of both single and batches of cell subtypes from the Comma-1D population. The supervised cellular populations identified from scRNASeq (P5, P6, P7, and P8), listed above in Table 2, were translated to populations identifiable via surface marker antibody expression. The Comma-1D cell line was thawed from frozen aliquots and cultured in DME media with Fetal Bovine Serum (FBS), Pen Strep (PS), Insulin, and Endothelial Growth Factor (EGF). To isolate specific populations, fluorescence-activated cell sorting (FACS) was performed utilizing cell surface expression of markers *Cd49f*, *Epcam*, and *Sca-1*. These isolated cell populations were then independently imported onto the Beacon 3500 chip. Each cell is tracked by the location identifiers pinned to each nanopen, allowing for cell type-related deconvolution downstream. By tracking cell types, it allows characterization of heterogenous batch culture at *t* = 0 (t_0_) and its effects on growth response. Cell samples from the original culture dish that were not sorted through flow cytometry were also imported onto the Beacon chip for growth analysis. Cells were cultured on-chip for 4 days with constant perfusion of Comma-1D culture media. Using the Beacon brightfield imaging mode (OEP), time-lapse images were captured every 12 h across the 22 Fields of View (FOV) composing the 3,500 independent nanopens, 311 of which contained at least one cell and 562 total cells penned. Growth was tracked across each nanopen. Each pen with cells (t_0_) was given a proliferation ratio identified by dividing the final cell count (t_96_) by the starting cell count in the pen. We identified no significant growth differences between the P5, P6, P7, and P8 population subtypes, as shown in Figure 6Bi. However, the population with the most considerable growth based on the calculated proliferation ratio was that which was not processed through the FACS pipeline. We observed a diminishing disparity between Comma-1D subtype-specific growth potential as cell counts in each nanopen at t_0_ increase, as shown in Figure 6Bii. Cell concentrations at t_0_ and t_96_ across all the cells imported in this experiment are presented in Supplementary Table S3.

To improve the image analysis pipeline, a pseudo-sorting platform was engineered to address the current shortcomings witnessed above by Comma-1D cell types processed through FACS. Comma-1D cells were incubated with immunofluorescent markers for *Epcam*, *Sca-1*, and *Cd49f* and then split into sorted and unsorted groups. The sorted group of cells was then processed through FACS to isolate the four subpopulations, as before. On the Beacon microfluidic chip, specific nanopens were dedicated to each subpopulation in the FACS sorted groups. These sorted cells were imported into the nanopens, while also controlling for cell counts in each nanopen. To investigate batch effects on growth, nanopens were allowed up to four cells at t_0_. The remaining nanopens were then allocated to the Comma-1D unsorted population of cells. A total of 562 cells were penned on this microfluidic chip. Using known marker expression parameters from previous iterations, we created a database of image intensities from the sorted population across all the imaging channels on Beacon. This database was used to then identify which subtype (P5, P6, P7, or P8) the unsorted cells represented and served as a pseudo binning tool within the Beacon platform. To visualize this, Figure 6C shows nine images from three separate nanopens. Each row represents the same nano pen at the same timepoint imaged on three fluorescent channels. Outlines were added around each nanopen; green boxes indicate the presence of fluorescence, and red outlines indicate low/no expression. In the top row of images in Figure 6C, the known P8 subpopulation isolated through FACS expressed high levels of *Epcam*, *Cd49f*, and *Sca-1*. This is the expected pattern of expression from the P8 population as identified from scRNASeq data and FACS. In row two of the images in Figure 6C, the unsorted cell A (U_A_) reflects the P8 subpopulation expression pattern by expressing all three markers at a detectable threshold. In contrast, unsorted cell B (U_B_) only significantly expresses *CD49f* and would therefore be categorized as a basal-like cell from the P5 subtype. P5 and P8 cells represent subpopulations that are likely to behave as “stem-like” and should therefore differentiate and self-renew.

This analysis facilitated pseudo sorting on Beacon and therefore eliminated the need for and the associated cellular stress from FACS. To further optimize this process, a custom MathWorks MATLAB script was developed with the workflow outlined in Figure 6D. Beacon chips are divided into 22 distinct imaging FOV, with all 22 FOVs composing the 3,500 nanopen chip when stitched. To develop the MATLAB script for automated cellular identification and pseudo binning, Comma-1D cells were incubated with immunofluorescent markers for *Epcam*, *Sca-1*, and *Cd49f*, as before. Without processing through FACS, 652 tagged cells were imported onto the chip. The target selection function on Beacon allows for controlled and reproducible image capture of each FOV in each fluorescent channel. To track cell response, a Region of Interest (ROI) is generated for each nanopen ID, as shown in Figure 6Di. By tracking nanopens we can compare populations within those isolated pens at various timepoints and imaging channels. Following this, cell detection was conducted using a circular Hough transform to identify possible cells by circularity within each ROI or nanopen. Figure 6Dii shows all regions detected prior to filtering. Filtering removed region centers not within a generated ROI for nanopens to remove false positives. Figure 6Diii displays filtered cell detection for a given FOV determined by measuring peak brightness at each possible cell location. A measurement reading of 65,535 consistently filtered false positives. Using the center matrix, each detected cell was then measured for fluorescence intensity in each channel on Beacon. This measurement value was normalized by dividing the average image intensity of all nanopen ROIs in that cell-free FOV. This normalization was necessary to compare fluorescence intensity between different FOVs as well as between sample chips as we observe variability in readings. Figures 6Div shows an example of normalized intensity readings for a subsection of a FOV in the TRED channel. Running this analysis across each ROI for each imaging channel generates a matrix of expression readings for each imported cell. To improve pipeline robustness for future assays, rather than employing a definitive threshold to differentiate expression with background readings, K-means clustering was used. Applying K-means clustering to each marker expression and then sorting clusters based on expression levels for each channel and protein serves as an automated binning of cells by surface expression. These bins were then leveraged across the channels to identify combinations that paralleled P5, P6, P7, and P8 subpopulations. Figure 6Dv shows a segment of a sample FOV with the text reflecting the unsupervised cell population each cell is predicted to belong to with a number “5” representing population group 5, or P5. Figure 6Dvi shows a scatter plot based on *CD49f* and *Epcam* expression, used to differentiate between basal-like and luminal-like cells within each colored cluster. To identify key pens of interest and increase efficiency in visualizing populations of interest, the script automated the export of images of the desired populations across channels. Figure 6E is an example of ROI images captured for some P8 identified cells and the measured intensities are shown as a heatmap. In both experimental runs, the maximum number of cells are penned from one 5 µl import. Of the 311 nanopens with penned cells in the FACS sorted preliminary experiment, 46 (14.8%) contained P8 sorted cells. The pseudobinning experiment had 176 nanopens with cells, from which 47 (26.7%) nanopens had with P8 identified. This accounts for an 80.4% increase in targeted cell culture count. Utilization of this pseudobinning helped address and prevent cell loss associated with traditional FACS sorting and furthered the image analysis toolset.

## Discussion

Utilizing scRNASeq data for gene expression analyses in conjunction with high throughput single-cell functional and proteomics data, we have developed a pipeline for both distinct population identification and validation. Through publicly available scRNA analysis tools, including Seurat and Monocle 3, we demonstrated the prediction of functional clusters within complex cell lines like Comma-1D. This heterogeneity highlights the need for further investigation into model systems, as well as high levels of intrinsic heterogeneity that must be considered when interpreting results, as they may confound conclusions of past and current studies.

The Comma-1D cell line is known to functionally differentiate in culture. It, therefore, served as a proxy to highlight the efficacy and resolution of the single-cell suite of methods discussed. Each cell type identified from the scRNASeq data lent toward a population to compare on the functional level for growth capacity. Within the eight subclusters identified from scRNASeq, there is observed partitioning of cells into luminal and epithelial groups. Each partition underwent deep characterization for further clarification on intra-partitional functional heterogeneity.

Luminal cells in the Comma-1D culture were identified as luminal progenitor and luminal differentiated cell types. These results reflect functional groups previously identified in mammary gland tissue, where (Cristea and Polyak, 2018) summarize luminal stem cell differentiation into either luminal progenitor, ductal, and secretary alveolar cells. Through DGEA and network analysis, a luminal progenitor and differentiated luminal population predicted to be secretory alveolar cells is identified within Comma-1D. Significant expression of immature luminal cell markers such as *Krt7* and *Ehf* are observed in the luminal progenitor cluster; however, we observed expression of differentiated cells markers such as *Krt8* and *Krt18* in that same population. Where (Wang et al., 2001) identified *Krt8/18* as markers of mature differentiated luminal cells within the prostate, we observed these markers associated with mammary luminal progenitors. These results underscore sources of variability in regard to disease type being investigated and cell line versus tissue gene divergence.

The presence of secretory cells with the absence of a non-secretory cluster within our population leads us to hypothesize two potential scenarios. One scenario is the cell line doesn’t fully differentiate into all cell types composing mammary gland function. The other hypothesis is that the non-secretory cells emerge from luminal progenitors at a later stage than alveolar cells, and at the stage of cell isolation and barcoding, the cell line had not yet reached this maturation point. The unclassified progenitor 1 cluster may represent secretory luminal cells that are differentiated and, therefore, are in later stages of the biological process needed to generate mammary gland cell types. Interestingly, the top DEGs in the progenitor 1 cluster are genes associated with keratinocytes and their role in cell-cell/cell-matrix interaction (*Lgals7*), structural components for hemidesmosome formation (*Col17a1*), and fibrous proteins for cellular support (*Krt5*) (Ali et al., 2021; Ho et al., 2022). Many of these functions mirror the needs of luminal cells within the mammary gland. Conserved gene expression within luminal cells not observed in the remaining population yield predictive information for patient disease prognosis or treatment. For example, *Wfdc2* encodes the He4 protein, which has already shown significant clinical benefit in monitoring and diagnosing ovarian cancer (Wei et al., 2016). Additionally, there has been a recent investigation into this marker’s application in breast cancer and, based on *Wfdc2* expression isolated in myoepithelial cells, we can predict that its efficacy in determining disease prognosis is more relevant for basal carcinomas than luminal (Chen et al., 2019).

Unlike the resolved heterogeneity in the luminal population, the myoepithelial cells identified by expression of *Acta2* were populated by clusters labeled myoepithelial 1–5 with limited functional granularity. The cnet plots illustrated in Supplementary Figure S3 visualize activated pathways in each population respective to the remaining cells in the same partition. Cells in myoepithelial 1 expressed *Mt1*, *Mt2*, and *Bnip3*, which are all direct activation of cellular response to metal ions or response to an inorganic substance (Koh and Lee, 2020). Metal ions such as Zinc are abundant in humans and play a role in the proliferation and differentiation of mammary epithelial cells (Han et al., 2020). Myoepithelial two cells significantly expressed *Pttg1* and *Cdc20,* both associated with reproductive processes in cellular development (Noll et al., 2015). Cells in myoepithelial 3 expressed unique markers such as *Pmepa1*, *Zyx*, and *Cdk1*. *Zyx* is a gene involved in actin reorganization for cell migration and EMT within the murine mammary gland (Mori et al., 2009). Myoepithelial 4 cells expressed genes such as *Acta2*, *Fn1*, and *Sparc,* which are linked to endothelial cell proliferation and wound healing pathways have been shown to respond within the mammary gland to facilitate healthy lactation function as well as controlling inflammatory response to stress (Ryman et al., 2015). The gene and pathway hits for this cell population indicate its interaction with the fibroblast cells, supported by their UMAP proximity. Similarly established cell line model IM-2, derived from the fourth mammary glands of pregnant BALB/c mice, demonstrated epithelial-fibroblast interaction in cell culture and its role in structural formation in culture with parallel functional differentiation (Reichmann et al., 1989). Cells in myoepithelial 5 expressed *Cstb*, *Ptges*, and *Txnrd1,* which are all genes linked to pathways associated with intracellular organelle lumen. These pathways are pivotal within mammary epithelial cells for the production of cytoplasmic lipid droplets, the precursor to milk fat globules (Chanat et al., 2016). While these pathway indicators don’t reveal exact functionality between the myoepithelial clusters, they generate an outline of heterogenous function within a cell line reflecting that of *in-vivo* cell populations. These results point toward a heterogenous myoepithelial population that shares the common function of organizing the mammary ductal network and aiding in milk transport while also retaining independent roles in the process.

Genes conserved in the myoepithelial clusters and lacking significant expression outside that partition provide markers for cell sorting and legacy experimentation. For example, *Lrp1*, which encodes cell surface proteins, provides a gateway to phenotypic cell identification and subsequent sorting for further downstream analysis. With this level of increased single-cell resolution data, we identified gene markers specific to clusters/subpopulations within the Comma-1D cell line (Supplementary Table S2). While extremely important for better resolving which systems can be modeled using Comma-1D, these markers can also be applied to patient single-cell data in parsing cell type populations in the tumor microenvironment.

When integrating the Comma-1D dataset with mouse mammary data from C57BL/6, concordant functional clusters were detected in both populations. The presence of luminal, myoepithelial, and fibroblast cells is identified in both samples. These findings further support the functional heterogeneity in cell lines that needs to be characterized with single-cell assays. Along with the concordant populations, the C57BL/6 data also indicated the presence of populations not initially found from the Comma-1D independent analysis. With the merged data, a ductal luminal population composed of 2,466 cells was identified with 102 (4.14%) of those cells only from Comma-1D. This population is characterized by markers such as *Csn3* and *Wfdc18* (Supplementary Figure S4D). The *Csn3* plays a role in stabilizing milk micelles, a key component of milk production during lactation (Komori et al., 2013). Both markers have been linked to ductal luminal cells that derive to secretory alveoli cells (Han et al., 2018). Through this integrated analysis, we confirmed functional heterogeneity observed in Comma-1D reflects populations detected in mouse mammary epithelium.

Orthogonal comparison of stemness markers and pseudotime analysis provides a metric for predicting clusters along with differentiation time points of the Comma-1D population. Monocle 3 was leveraged for pseudotime analysis, where we identify cellular partitioning reflective of the cell clustering from the Seurat analysis. The Comma-1D dataset was reprocessed for dimensionality reduction on Monocle, after which the differentiation trajectory was inferred, and gene expression was plotted to track changes over pseudotime. Supervised pseudotime analysis identified the luminal progenitor population as a possible origin population in one partition of the dataset. The verification of gene-based functional predictions by entropy score validates this assay as a tool for screening progenitor-like cells, particularly beneficial when investigating an uncharacterized population or dataset.

There have been abundant investigations into stemness markers within the mammary gland (Yang et al., 2017; Zhou et al., 2019; Engelsen et al., 2020). These efforts highlight potential co-expression protein combinations that may be utilized for specific stem-like cell populations isolation, including *Lin* ^*-*^ *CD29* ^*high*^ *CD24* ^*+*^, *CD44* ^*+*^ *CD24* ^*-*^ *Lin* ^-^, and *ALDH1* ^*+*^ *Sca-1* ^*High*^ (Yang et al., 2017). Despite abundant evidence for the expression of stemness markers within this cell line, there remains no established protocol to define the extent of cell differentiation. Identifying stem cells from genomic profiling is an assumptive process, but any molecular discoveries can now be phenotypically validated using custom pipelines on real-time cellular manipulation platforms such as Beacon.

Intersecting published stemness markers within the mammary gland with our scRNASeq dataset identified *Epcam* ^*+*^ *, Cd49f* ^*high*^ *, and Sca-1* ^*high*^ markers of interest for further investigation. All populations had a subset of cells, P8, expressing all markers except for fibroblast. As we do not expect differentiated fibroblasts to play a role in stemness, this further supports the likelihood that these markers can be utilized in identifying cells that may have stem-like properties. The P8 subpopulation is of interest due to its “stem-like” properties, where *Sca-1* is a key identifier of Hematopoietic Stem Cells (HSCs) (Morcos et al., 2017). While the P8 population presents the most potential for stemness based on known marker expression, investigating the other populations lacking expression of one or more of the identified genes also yielded functional identification regarding differentiation and proliferation capability. Determining functional differences from just scRNASeq data is not direct; however, we can pair the inferences made from this pipeline to observe and validate phenotypic differences between cell populations. Through the linkage of high throughput single-cell data generation pipelines across multiple cellular variables, we were able to gain deeper insight into single-cell functional heterogeneity.

Platforms like Beacon provide a high throughput single-cell testing method to address the emerging need to further investigate and validate the predicted phenotypic variants within a population determined from assays such as scRNASeq (Maddaly et al., 2017; Duarte et al., 2018; Kapalczynska et al., 2018; Sachs et al., 2018; Takebe et al., 2018; Xu et al., 2018; Xia et al., 2019). The Beacon instrument contains a 3-axis platform with four nests for cell culturing in four isolated chips. An imaging cube is fixed above the nest for imaging in 4 fluorescent channels: FITC, CY5, DAPI, and TRED. Parallel to conventional organoid growth protocols, optimizing Matrigel concentration, cellular penning parameters, media conditions, and fluidics chip conditioning was required (Le Gac and van den Berg, 2012). While cell growth has been selectively demonstrated on this platform for Comma-1D, multidimensional variability can activate distinct cell pathways and requires iteration for each biologic model (Beaumont et al., 2022).

scRNA sequencing provides high resolution data for subpopulation identification. We used this data to predict functional subgrouping within a population to understand the sources of disease progression and metastasis. Through scRNASeq and protein-level comparisons, the 4 populations in Table 2 were identified based on the expression of *Epcam, Cd49f,* and *Sca-1*. Comma-1D cells from culture were sorted using FACS for surface expression of the given markers and imported onto a Beacon chip along with unsorted Comma-1D cells from the same culture. Distributions of t_0_ and t_f_ counts are outlined in Supplementary Table S3 and a summary of nanopen and cell counts in this experiment is outlined in Supplementary Table S4. We observed significant growth in the unsorted population compared to the sorted P5, P6, P7, and P8 populations. While FACS allows high throughput sorting, current sorting systems have been shown to result in cellular stress, as described above, and diluted concentrations of rare cell populations (Sauvat et al., 2015; Llufrio et al., 2018). This result may be due to either flow-based sorting neglecting key cells of interest that skew proliferation or effects on cell expression due to the sorting process that inhibited proliferation. In either case, we believe that flow sorting compounded with the microfluidic and OEP cell importing of Beacon resulted in cell stress activation that potentially inhibited cell growth on-chip. These factors become more significant when processing patient samples where cellular stress and rare cell loss are already impacted (Reuben et al., 2015). As the initial count of cells at t_0_ increases, we observe a depreciating disparity between sorted and unsorted cell growth. This could be due to microenvironment changes that initiate cell adherence and growth from intercellular signaling. Further investigation will be performed to gain insight into defining cell subpopulations and their individual delta on the impacts of the microenvironment and cell aggregate growth.

Fromthe preliminary growth data described above, we observed that this cellular stress reduced cell growth during the Beacon culture period. To address this, we leveraged the imaging channels on the Beacon instrument to identify populations of interest from unsorted Comma-1D cells that were incubated with fluorescent markers of *Epcam, Cd49f,* and *Sca-1*. The three fluorescent imaging channels were paired to independent markers, and each nanopen was imaged on each channel at 12 h interval timepoints. After subtracting expression readings with baseline values determined from empty nanopen readings and normalizing measurement readings from background, we generate expression vectors for each cell in each nanopen across imaging channels. These vectors allow pseudo-binning of unidentified cells into populations of interest. Since our P8 cells of interest are rare in the population, utilizing this pseudo-sorting capability also bypassed processing steps such as centrifuging that are conventionally necessary for FACS, further reducing cell loss and improving cell viability. After numerous iterations on Beacon, we refined our import and culturing protocol resulting in reliable cell adhesion and growth from a subset of cells. The MATLAB image analysis script allowed bypassing of FACS, preventing loss of rare cells and reducing cellular stress. Similar analyses of novel cell marker expression and single-cell growth data will likely yield the highest throughput analysis of cell subtype effects on cancer malignancy potential. The built-in cell analysis platform could not be used in this study due to compromised control on time point comparisons, as well as no available sorting algorithm for penned cells. The custom method presented here can be used more generally for high throughput separation and image analysis to focus on pathologic or other cells of interest.

Celllines are used across disciplines and have unknown levels of subpopulation variability, which can alter the ability to draw definitive conclusions from *in vitro* studies. In the case of therapeutic development, *in vitro*, human cell lines have been key systems for predicting both efficacy and toxicity of drugs (Allen et al., 2005). For the Comma-1D line, which here was identified to contain two cell partitions composed of eight clusters, it is not unlikely that certain subtypes would respond differently to therapy than others. Moreover, it is a cell line with a background of known differentiation, so we may see a more drastically heterogeneous population as compared to other breast model lines (Vachon and Beaulieu, 1992; Vranic et al., 2011). As the standard of oncology treatment moves toward targeted therapies, our understanding of model systems used as the first line of testing needs to be improved through higher resolution characterization (Levine, 2000; Tripathy, 2002; Arruebo et al., 2011; Nounou et al., 2015). Further scRNA investigation paired with phenotypic observations can provide the needed level of deep insight into cell populations used for these types of critical studies.

We present here an assay for identifying and monitoring functional characteristics of single cells at a high throughput level. Using a custom pseudo sorting script, we identify cell subtypes across up to 3,500 pens on the Beacon platform. Each of the pens and respective cells were tracked for growth response. By utilizing the automated script, we also prevented the loss of rare cells typically observed in flow sorting and reduced cellular stress allowing for improved growth. With this process established, we laid the foundation for the application of these analyses to varied cell lines modeling a wide variety of disease states. Furthermore, we can apply this pipeline to patient samples to diminish processing times and the impact of sample handling currently applied by FACS. By reducing the time between sample collection and functional assay, we will preserve sample quality and obtain a more accurate understanding of the patient’s disease state. Ultimately, the development of high throughput single-cell multi-data assays can resolve the contribution of various tumor microenvironment components to disease pathogenesis and tumor metastasis. This information can be leveraged for high throughput single-cell assays to quantify therapeutic response, classify differentiation heterogeneity in generated models, and validate the conclusions identified from single-cell sequencing.

## Data Availability

The datasets presented in this study can be found in online repositories. The names of the repository/repositories and accession number(s) can be found below: https://www.ncbi.nlm.nih.gov/geo/, GSE182589.
