# Supplementary figures and images for: Integration of Single-Cell Transcriptomics With a High Throughput Functional Screening Assay to Resolve Cell Type, Growth Kinetics, and Stemness Heterogeneity Within the Comma-1D Cell Line
Source: Front Genet. 2022 Jun 14:894597. doi: 10.3389/fgene.2022.894597 (PMC9237515; doi:10.3389/fgene.2022.894597)

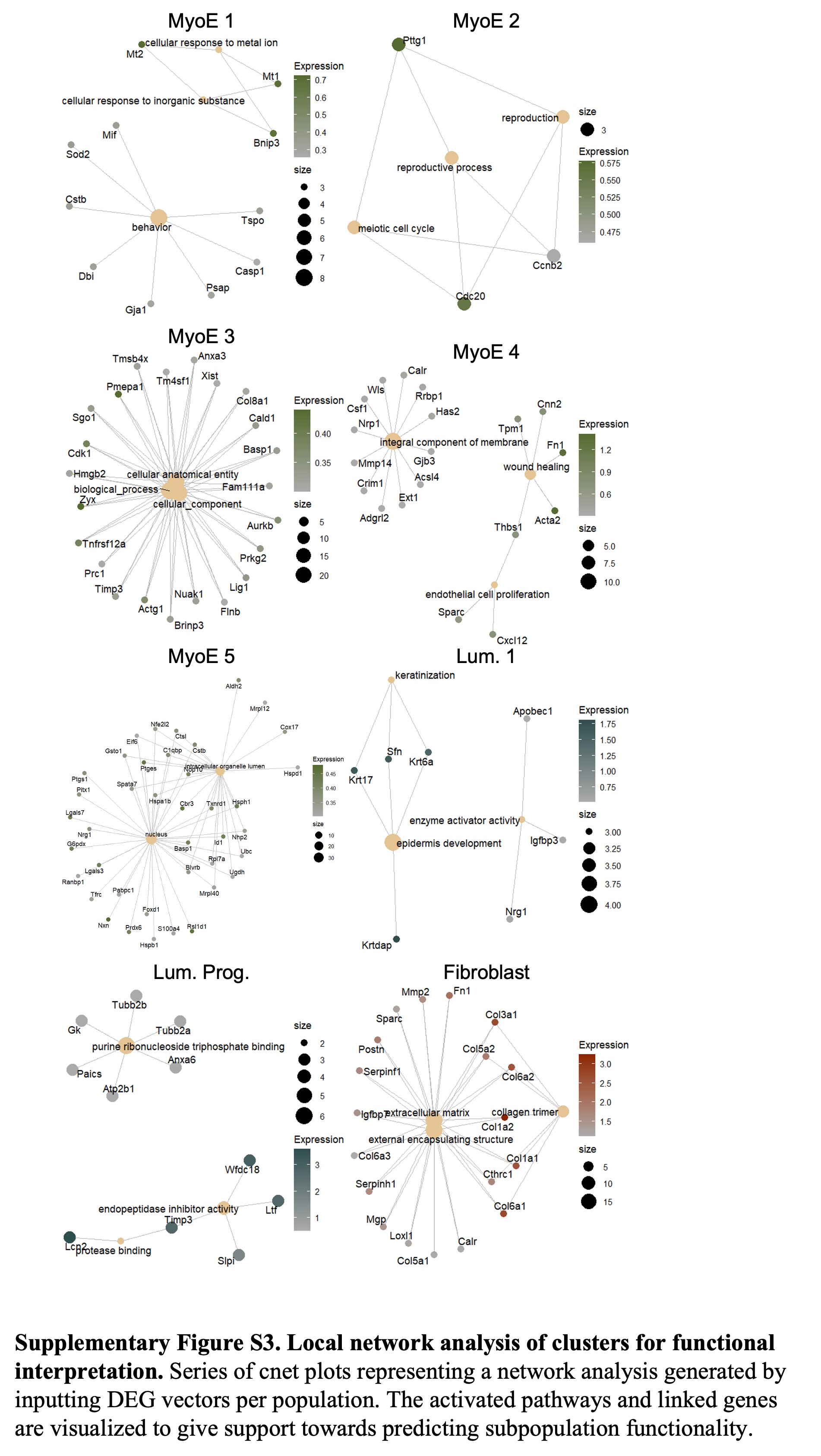

Supplement: Supplementary file 1 [file Image3.JPEG]

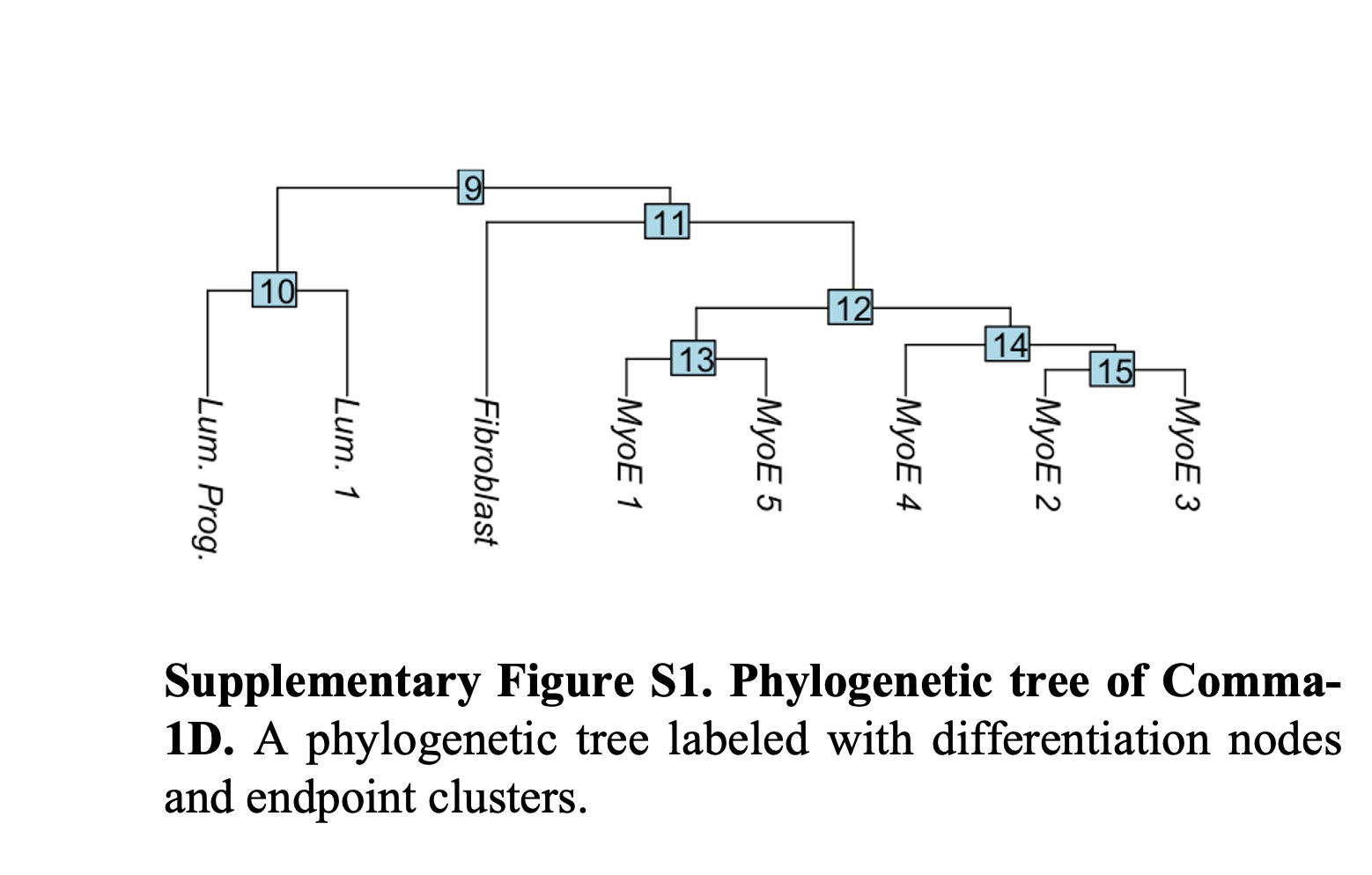

Supplement: Supplementary file 2 [file Image1.JPEG]

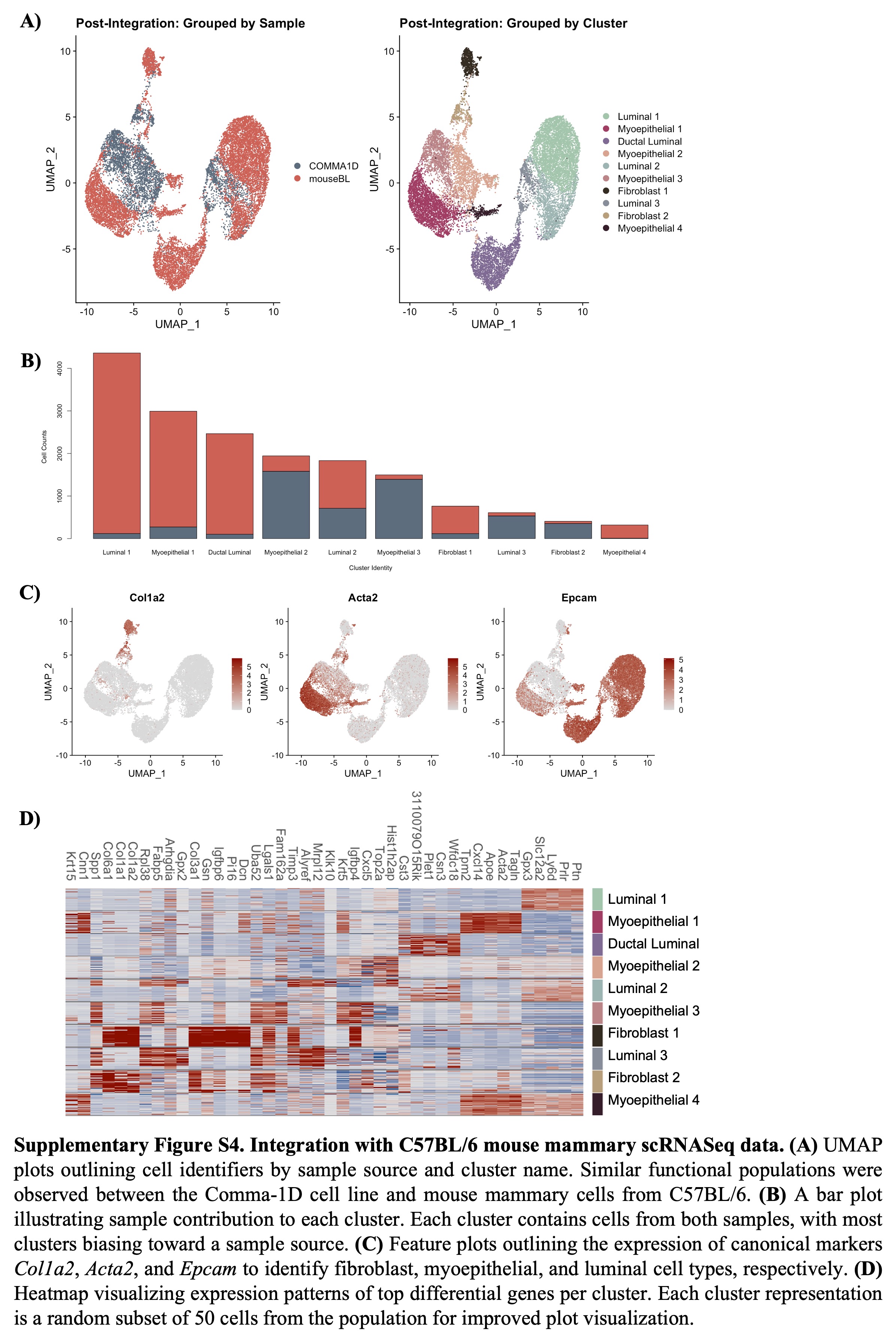

Supplement: Supplementary file 3 [file Image4.JPEG]

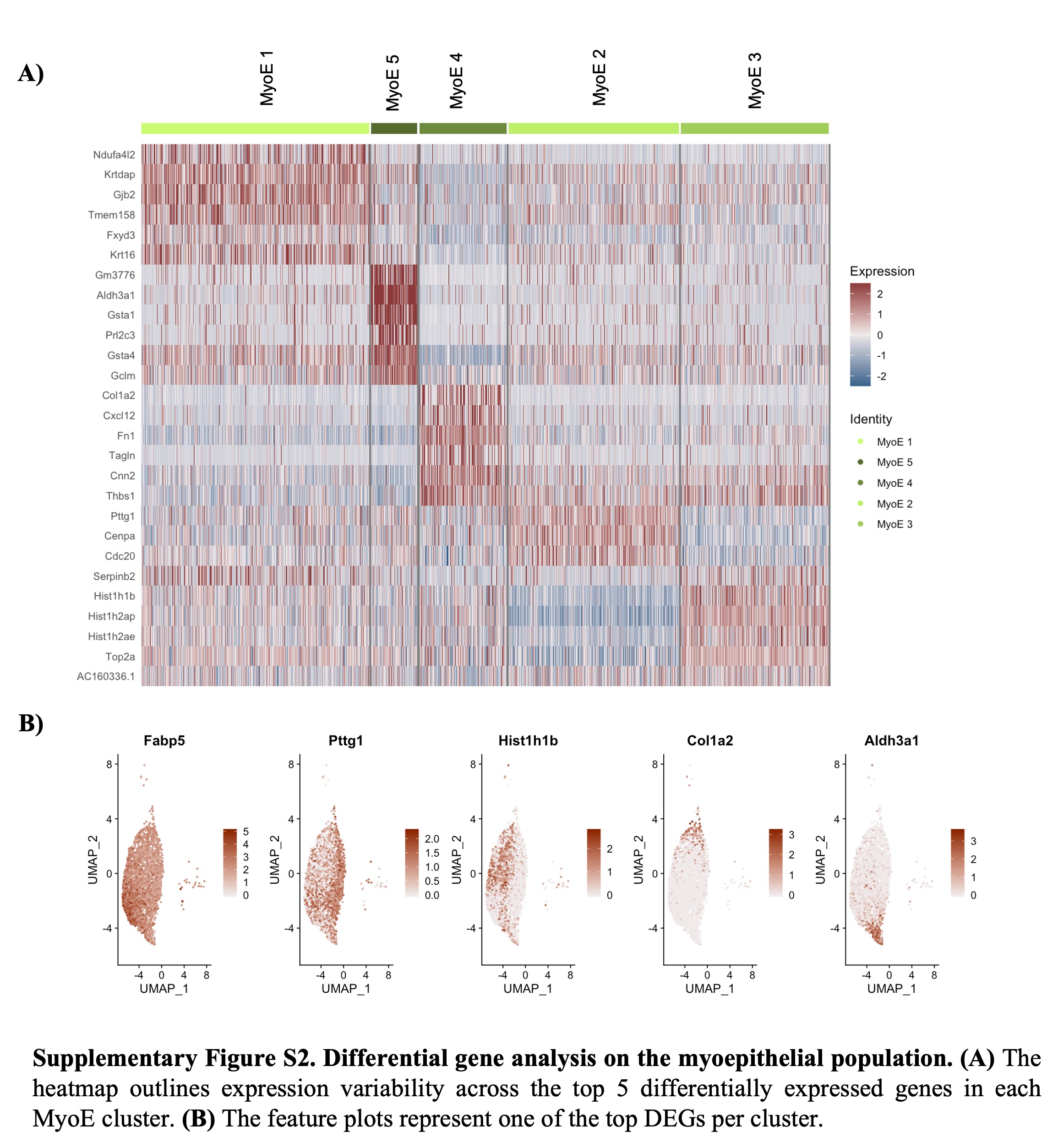

Supplement: Supplementary file 4 [file Image2.JPEG]

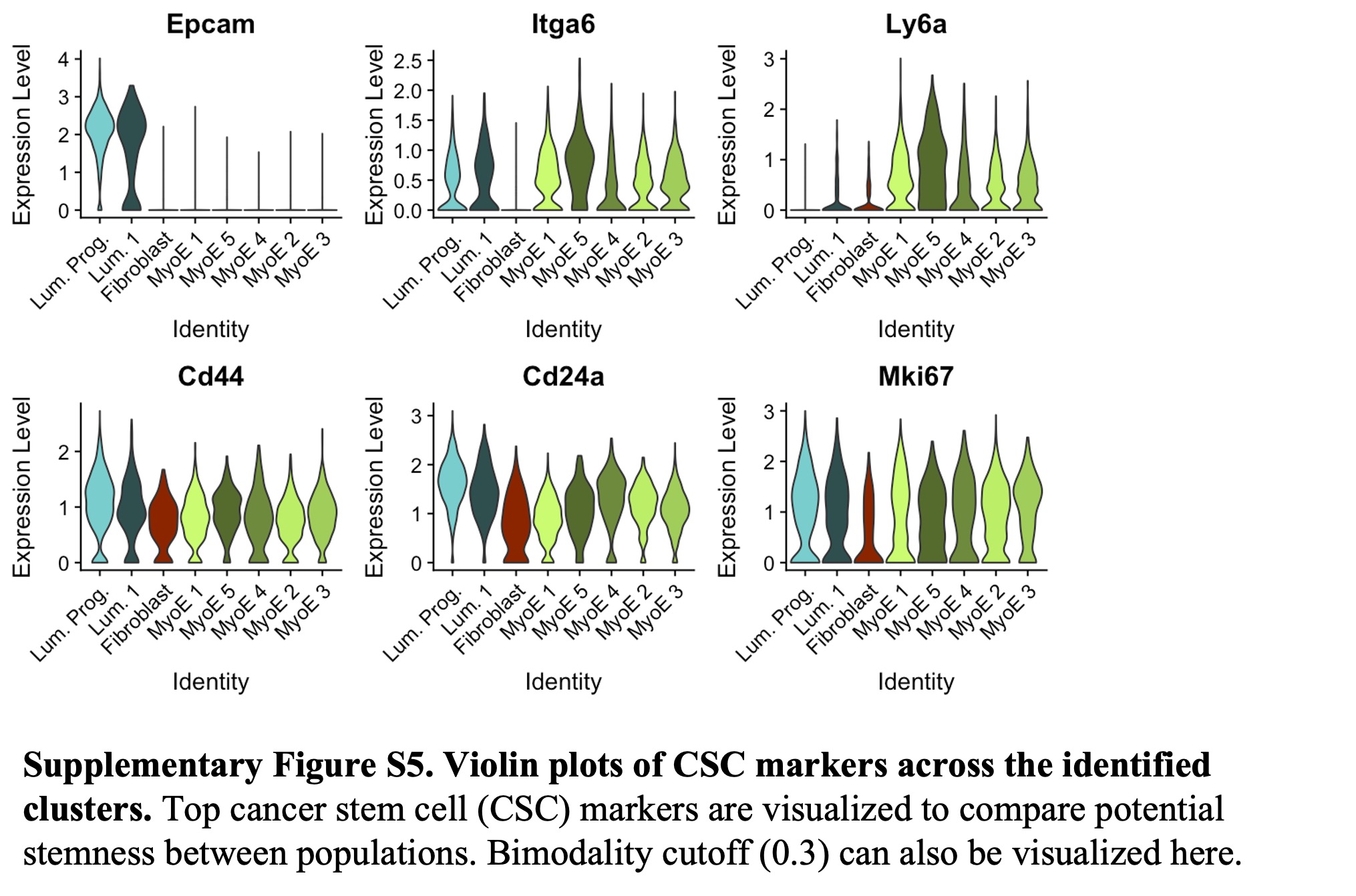

Supplement: Supplementary file 5 [file Image5.JPEG]
